# Quantifying gaze and mouse interactions on spatial visual interfaces with a new movement analytics methodology

**DOI:** 10.1371/journal.pone.0181818

**Published:** 2017-08-04

**Authors:** Urška Demšar, Arzu Çöltekin

**Affiliations:** 1 School of Geography & Sustainable Development, University of St Andrews, St Andrews, Scotland, United Kingdom; 2 Department of Geography, University of Zurich, Zurich, Switzerland; The University of Melbourne, AUSTRALIA

## Abstract

Eye movements provide insights into what people pay attention to, and therefore are commonly included in a variety of human-computer interaction studies. Eye movement recording devices (eye trackers) produce gaze *trajectories*, that is, sequences of gaze location on the screen. Despite recent technological developments that enabled more affordable hardware, gaze data are still costly and time consuming to collect, therefore some propose using mouse movements instead. These are easy to collect automatically and on a large scale. If and how these two movement types are linked, however, is less clear and highly debated. We address this problem in two ways. First, we introduce a new movement analytics methodology to quantify the level of dynamic interaction between the gaze and the mouse pointer on the screen. Our method uses volumetric representation of movement, the space-time densities, which allows us to calculate interaction levels between two physically different types of movement. We describe the method and compare the results with existing dynamic interaction methods from movement ecology. The sensitivity to method parameters is evaluated on simulated trajectories where we can control interaction levels. Second, we perform an experiment with eye and mouse tracking to generate real data with real levels of interaction, to apply and test our new methodology on a real case. Further, as our experiment tasks mimics route-tracing when using a map, it is more than a data collection exercise and it simultaneously allows us to investigate the actual connection between the eye and the mouse. We find that there seem to be natural coupling when eyes are *not* under conscious control, but that this coupling breaks down when instructed to move them intentionally. Based on these observations, we tentatively suggest that for natural tracing tasks, mouse tracking could potentially provide similar information as eye-tracking and therefore be used as a proxy for attention. However, more research is needed to confirm this.

## Introduction

Humans experience the world through a variety of senses, among which vision plays a dominant role [1,2]. Visual processes can be bottom-up (environmental features attract our attention), or top-down (we consciously decide where to look); but in either case, human visual behaviour is often linked to attention [3]. Therefore, studying eye movements can reveal valuable insights into how people think, especially when performing visuo-spatial tasks.

Eye movements are recorded using specialized eye tracking devices that register a series of gaze positions on a display. Through eye tracking, we obtain temporal sequences of gaze locations, or the so-called *gaze trajectories*. Raw gaze trajectory data are typically aggregated into *scanpaths*, i.e., sequences of fixations (locations where eye is ‘fixed’ for a brief period of time) and saccades (quick movements between fixations) [4]. A number of methods have been proposed to analyse aggregated scanpaths (gaze plots, ‘heat maps’, methods to assess consistency of scanpaths, aggregation of scanpaths into areas of interest, etc., see [5] for a comprehensive review); however, the raw gaze trajectories, which represent the actual eye *movements*, have been largely neglected.

In recent years, eye tracking devices are becoming more affordable [6] and new technological solutions appear to allow crowdsourcing eye tracking [7]. Nonetheless, eye tracking experiments are still complex, costly and time consuming, require dedicated devices, and despite the recent developments, currently, reliable eye movement data can only be collected one person at a time [8–10]. Therefore, as an alternative (or, in addition to eye tracking, especially those with lower accuracy such as web-cam based eye tracking without integrated active sensors), studying mouse movements has been suggested, as mouse movements can also express certain cognitive processes during visuo-spatial task solving [11]. Collecting mouse data has practical advantages as most devices are already equipped with a mouse (i.e. they not require additional specific equipment), and mouse data can be collected automatically as a background process for a large number of participants in parallel. Using mouse trajectories as a proxy for gaze trajectories has been proposed by Huang et al. [12], and various studies that collected and used mouse movements seem to support the idea that mouse movements might be representative of eye movements to some degree [13–15].

The proposition that the mouse and eye movements would have certain similarities when we work with visuo-spatial displays is not surprising. There is ample evidence demonstrating the connection between hand motion, vision and mental processing, even though the nature of this connection is only just beginning to be explored [16–18]. In Human-Computer Interaction (HCI), the interaction between gaze and mouse has been investigated for tasks that involve reading information from displays [12,19]. Particularly relevant to our study, Bieg et al. [20] investigated the connection between the gaze and the mouse for a set of visual search and selection tasks on graphic displays. They tested the commonly used assumption that the target object on the screen is fixated with the gaze slightly before it is selected by the mouse pointer. They found that this is generally the case, but observe an interesting effect that if the location of the target object on the screen is known, the pointer follows faster than expected, and its movement is initiated before the target has been fixated with the gaze. In addition, some participants in Bieg et al. [20] study employed the pointer as an active reference point for keeping track of the search path. Such referencing behaviour has also been previously observed in web browsing [19]. This type of effects (e.g. mouse following the gaze and using the pointer to keep track of the search path) may be of particular interest for spatial and geographic tasks, such as route tracing on a map display. However, this connection is underexplored, perhaps for the lack of appropriate spatio-temporal analytical methodology to quantitatively describe the connection between the two respective movements.

In this paper, we propose *a new spatio-temporal methodology for quantifying the connection between the gaze and mouse movements*, which we base on contemporary developments from computational movement analysis and visualisation [21]. To evaluate and validate our new methodology, we devise an experiment which mimics a simple spatial task commonly performed by users of geographic displays–route tracing–and in which we synchronously collect gaze and mouse data. The experiment is designed to generate data with a known delay of the mouse behind the gaze and vice versa for control purposes, as well as data on how route tracing is done in a natural manner.

Our new analytical methodology considers the two types of movement (gaze and mouse) as spatio-temporal phenomena. Eyes move in high speed, almost discrete, jumps (saccades) interspersed with longer, almost fully stationary, fixations, while mouse movement is relatively smooth and continuous. Consequently, eye and mouse trajectories have physically very different movement characteristics and the two types trajectories are difficult to compare to each other [22]. Another important difference between the two movements is that mouse movements are normally under conscious control, while eye movement are only rarely performed consciously in a scenario such as ours (i.e. for route tracing), and only so when specifically instructed, e.g. for using gaze-controlled interfaces [23]. However, the two movement types are connected through the following three facts:

1. Both movements (gaze, mouse pointer) can be represented as trajectories, i.e. time series of observed locations on the screen.
2. The two movements, and consequently the two trajectories, are co-located in space and time, i.e. the movement space is the same (a 2D computer screen) and movements occur simultaneously.
3. Both movements are generated by the same cognitive process, in which the participant visually investigates a spatial stimulus on the screen, and performs a spatial task (in our case route tracing).

Based on these three facts, we propose that the interaction between eye and mouse movements can be quantified by analysing the level of co-occurrence of the two trajectories in space and time. We propose investigating the level of this co-occurrence through 1) time series analysis of distances between the gaze point and the mouse pointer and 2) quantification of dynamic interaction using a volumetric 3D representation of each type of movement–the space-time densities [24], combined with a 3D generalisation of change detection methods from remote sensing [25].

The remainder of this paper is structured as follows: in the related work section, we introduce relevant terminology, then review eye and mouse movement studies in HCI and methods for evaluating dynamic interaction of moving objects. This is followed by a description of our experiment and the hypotheses about the level of gaze and mouse interaction that we expected to see in different visual tasks. We then introduce and define our new analytical methods, conduct method comparison with dynamic interaction indices from movement ecology, perform a sensitivity analysis, and finally conclude with experiment results and a discussion of wider implications.

## Related work

### Terminology

Human visual field is usually separated into three different regions: foveal, parafoveal and peripheral [26]. The fovea is the region in the centre of the retina that has the highest visual resolution and is densely covered with cones (photoreceptor cells that capture full colour and high resolution detail). Foveal region has a diameter of about 0.3mm and corresponds to the central 2° of the visual field [27]. It is surrounded by a belt with the highest density of the second type of photoreceptors, rods (not sensitive to colour, but very sensitive in dim light conditions and motion). This area is the parafovea, which extends to 5° of the visual field. Foveal vision has the highest visual acuity and spatial contrast sensitivity [28], while parafoveal vision has a higher temporal frequency resolution and is important in sequential tasks, such as reading (where the parafoveal pre-processing influences the efficiency of foveal vision) [29]. Vision beyond parafovea is called peripheral vision. Peripheral vision plays an important role in monitoring for change in the visual environment and is sensitive for flicker and temporal change [30], however, arguably, might be less important in interacting with a (static) spatial display.

To bring the target of interest into the foveal area on the retina, the eyes need to be able to move. There are three main types of eye movements: saccadic movements, smooth pursuit and vergence movements [3,30]. We are particularly interested in saccadic and smooth pursuit movements, while vergence movements, which adjust the two eyes in coordination to track a target through different levels of depth, are of less relevance for our experiment.

*Saccadic movements* rotate the eye to fixate the visual axis on the target of interest. These movements are fast, jump-like, and interspersed with periods of relative stationarity, when the eye rests on the target–the so-called fixations. Fixations last between 80 and 400 milliseconds, while saccades take between 20 and 180 milliseconds, depending on the angle traversed [5, 9]. Saccades are ballistic, meaning that they are pre-programmed in the moment when the brain decides to switch attention to a new location and cannot be modified once the movement has been initiated. During a saccade, a masking mechanism stops visual processing (i.e. we see very poorly while a saccade is in progress), which is called ‘saccadic suppression’ [5, 9]. During a fixation, the eye appears almost stationary, but is in fact slightly jiggled by two smaller types of movements: the slow irregular drift and the rapid irregular tremor. These irregular micro-movements introduce small changes to the level of stimulation of each individual cone, to prevent adaptation to an unchanging light signal (if this adaptation occurs, the cones stop registering colour and all we see is grey). Saccades normally operate below visual awareness, but can also be made consciously [26,30].

During the *smooth pursuit* movement, the eye is fixated on a moving target and tracks the target across the visual field with the same speed as that of the target [31]. This type of eye movement is initiated with an open-loop step, which is a ballistic movement of the eye onto the moving target. The remainder of the pursuit occurs within a closed-loop, where the angular velocity of the target is nearly equal to the angular velocity of the eye. This type of eye movement is under voluntary control and can be performed consciously.

Foveal vision is the primary mechanism for *attention*, that is, it allows us to select an object or a location in the visual field for analysis, while the objects that are not in the foveal region receive less processing. This type of attention is called *direct* or *overt* attention and it inevitably follows the movement of the eyes and the sequence of fixations. In addition to this, it is also possible (although more difficult) to notice objects outside the foveal area, without moving the eyes. This is the *indirect (covert) attention*, and there is behavioural and physiological evidence that information obtained through the mechanism of covert attention determine the next fixation [30]. Smooth pursuit movements are also tightly coupled to spatial attention to the tracked target, while the surroundings, including motion signals in the same direction, are less well processed once the closed-loop pursuit is established [32,33]. Eye trackers capture data mainly on overt attention through identification of foveal fixations in the gaze trajectory. Mouse movements in our experiment, at least with some of the tasks, could be indicative of indirect (covert) attention through parafoveal and/or peripheral vision.

### Coordination of the eye and the mouse movements

Recent neurophysiological advances show that manual dynamics are inherently coextensive with mental dynamics [16,17]. In other words, hand movements can provide information on internal cognitive processing of users who use a pointing device, such as a computer mouse [23]. It is, therefore, not surprising that mouse tracking has become popular for observing and inferring users’ behaviour in various tasks.

As we are concerned with development of new methodology for evaluation of gaze and mouse interaction, in this section we consider mouse tracking studies from a methodological point of view. Mouse data are generated by continuously sampling the position of the mouse pointer on the screen, and we focus on studies that treat these data as trajectories. Freeman et al. [13] have recently introduced a software called ‘MouseTracker’ which implements a set of trajectory measures for mouse trajectories. These measures include; calculating representative mean trajectories, time and space re-scaling, measures for spatial attraction and distributional analysis and measures of complexity, which measure the amount of direction flips. All of these measures are used on raw mouse trajectories, that is, trajectories where only sequences of mouse locations are considered, without any additional information. Another software, specialised to slide designs, OGAMA, (the OpenGazeAndMouseAnalyzer [34]), provides functionality for traditional eye tracking analysis (e.g. identification of fixations, analysis of scanpath similarity, generation of attention maps, etc.), but only limited tools for analysing mouse movement.

Another study that uses raw mouse trajectories is by Tahir et al. [15], who investigated the similarity of mouse movements on the screen across a group of participants. They use two geometrically-defined similarity measures (close destination and route-based similarity), and evaluate two different trajectory clustering algorithms with these two measures: the OPTICS algorithm and a density-based clustering. Their evaluation dataset is from an experiment using a geographical web interface, where users were asked to perform typical spatial tasks (route planning, visual search, etc.). In a previous study [14], the same authors consider semantically enriched mouse trajectories, that is, trajectories of mouse positions, where additional information such as mouse clicks, mouse hesitations, mouse speed and mouse interaction mode (zooming and panning) is recorded. They introduce a set of visual and computational tools to deal with this type of data.

Linking mouse movements to eye movements by evaluating the dynamic interaction of their trajectories as we propose has not been previously done. However, hand-eye coordination has been a long-standing topic in psychology and has conceptual similarities to our thinking. For example, Ballard et al. [35] investigated hand-eye coordination on a series of sequential tasks while Inhoff et al. [36] performed a study on copytyping (typing from text) using eye tracking and tracking the timing of actual typing (pressing of the buttons) as a proxy for hand movement. Interestingly, a similar eye and hand movement experiment on copytyping was performed already in the 1930s [37], where eye movements were recorded with a special camera and the timing of these movements obtained from the film that was used to annotate the original text with times of fixations. In addition to capturing the eye movements, the film recorded moments in which the carriage of the typewriter reached one of the contact points, of which there were six in each row of typed words. When this occurred, a clever system of electric time-keeping momentarily turned off the light, which was recorded on the film. These moments of no light were then annotated in the copied text as a proxy for times of hand movement and compared with times of eye fixations.

More recently the connection between the eye and hand movements has been explored in context of using the mouse as the computer input device [38]. This has mostly been done for web browsing and web search tasks [19], typically using a combination of basic mouse metrics (number of clicks) and standard eye-tracking metrics (number of fixations, fixation duration, time spent on task, etc.) to evaluate navigation in a web mapping tool [39]. At a more basic level than evaluating a specific web application, Bieg et al. [20] measure the level of coordination between eye and mouse pointer in simple tasks (visual search and selection of graphic entities on the screen), as already described in the introduction.

### Movement analysis and measures for dynamic interaction

The methodological question that we are addressing in this paper is how to quantify the connection in space and time between two moving ‘objects’, i.e. the positions of the gaze and the mouse pointer on the screen. Quantifying such connections is a long-standing problem in scientific disciplines that analyse movement data. While our approach has, as far as we are aware, not been applied in eye tracking, it is common in other disciplines, in particular in movement ecology, which investigates animal movement [40]. There, this connection is called *the dynamic interaction* (sometimes also relative motion between two objects, or association or correlation of two moving objects) and is defined as the level of inter-dependency between two moving individuals [41]. Analysing dynamic interaction allows ecologists to categorise various types of movement behaviour, thus learn more about the animals and their interactions with each other as well as with the environment. This analysis can be done either between individuals, where patterns such as grouping, following, frequency of encounters or avoidance/attraction are of interest [42,43]; or between co-located species, looking at predator/prey behaviours, avoidance or chasing [44,45].

There are many analytical methods for evaluation of dynamic interaction. Some use geometric properties of trajectories [46,47], others identify clusters of sub-trajectories [48] or look at temporal evolution of groups of individuals that meet, join, move together and part [49]. One of the well-known families of dynamic interaction methods are the ones that use the principles of time-geography, which is based on the inherent interdependency of space and time [50]. Time geography defines the conceptual space (and a visualisation approach) of a Space-Time Cube (STC, [50]) to illustrate this interdependency. The STC is a 3D space where the bottom two dimensions represent geographic space (or in a more general sense, the space within which movement occurs), and the third dimension represents time. While over forty years old, in recent years its popularity has grown, both in its native discipline, GIScience [51], but also in related areas, such as Information Visualisation [52]. The STC has also been used in HCI to display and evaluate eye-tracking trajectories [53–55].

Movement data can be represented within an STC either directly, that is, as trajectories, or in an aggregated volumetric form. Trajectories are modelled as monotonically increasing polylines within the 3D space (since movement is always progressing in time, trajectories cannot ever stay static, i.e. horizontal at one temporal level, nor can they ever turn downwards). Volumetric representation is by space-time densities, which are generalisations of 2D kernel densities to 3D [24, 56]. These 3D densities are used for visual identification of frequent patterns in movement, such as spatio-temporal hotspots. We use them to define the *field of influence* in space and time around a particular trajectory.

Another approach to volumetric representation of movement is the space-time prism [57], which is an ‘accessibility volume’ in an STC between two movement points. These prisms are not prisms in a strict mathematical sense, but are defined as intersections of two accessibility cones, each of which has its peak in one of the two end points of one movement segment. The cone of the start point is oriented towards the future and the cone of the end point towards the past, both having the movement segment as the central axis of the cone. Both cones stretch into infinity and represent the volume in the STC, which the moving object could access in the future or in the past, given its velocity and direction of movement at the respective peak point. The intersection of these two cones represents the accessibility volume around the movement segment and is called the space-time prism.

Projecting space-time prisms onto the 2D space of movement (i.e. the base space of the STC) results in the so-called Potential Path Area (PPA), that is, the ellipse within which the movement could have occurred between two observed locations, based on physical characteristics of movement [57]. Intersecting two PPAs has been used in several methods for dynamic interaction, both for animal movement [58] and for human movement [59]. Intersecting two PPAs creates a so-called *social-interaction space* [60], which represents the boundary of the area within which direct inter-individual interaction between two moving objects can occur.

We combine the 3D volumetric representation of movement in an STC (representing the movement space of gaze and mouse on the screen, and time) with the idea of representing dynamic interaction of eye and mouse as intersection of the two respective volumetric fields of influence. This intersection is similar to the idea of social-interaction space as an intersection of space-time prisms via PPAs [60]. We, however, use space-time densities around polylines rather than space-time prisms, and we also perform the intersection in the original 3D STC space rather than projecting the volumes onto the base 2D movement space. The extent of intersection in the entire volume is then used to assess the level of dynamic interaction between two moving objects (in our case, the gaze and the mouse pointer).

## The experiment

We designed and conducted an eye-tracking experiment coupled with mouse-tracking to test and validate our methods. In this section, following the standard practice, we present the necessary information on the participants, the apparatus, the design of the experiment, and the procedure. Furthermore we elaborate on the data (and their pre-processing) that we obtained from the experiment. The eye-tracking experiment was approved by the University Teaching and Research Ethics Committee (UTREC) of the University of St Andrews (approval no. GG9914). Participants were asked to give written informed consent and the consent form was approved by UTREC.

### Experiment design and hypotheses

Our experiment had a within-subject factorial design with two main factors: task type (natural tracing/eye-first/mouse-first) and geometric shape type (circle/rectangle/flower/star) where all participants executed all tasks with all stimuli. All (eleven) participants executed a total of twelve tasks. We provided the four geometric shapes as stimuli and instructed the participants to trace the borders of each of these shapes in three specific ways. The task type was chosen to mimic route tracing, which is common when working with geographic displays on the screen, such as for digitising spatial features, or for measuring distances along a route. For this experiment we have chosen the simplest possible route tracking task: shape tracing, where the only complexity was introduced by the geometric form. This is of course much simplified from the real route tracing on a map, where attention and thus gaze may be distracted by a number of features, such as the complex structure of the route, presence of text and presence of landmarks. However, one of the aims of this experiment was to generate a data set of real gaze and mouse movements that were as free from distractions-based noise as possible, so that we could use it to demonstrate how our new methodology works. Therefore, for maximum control on the visual stimuli, we used the simplest possible tracing tasks. We plan to evaluate real route tracing on real and complex maps in a future experiment.

We had two goals in mind when we designed the experiment: to obtain a preliminary investigation of the visual behaviour of participants when tracing a route on the screen, and at the same time to produce a data set of gaze and mouse trajectories that was expected to display specific types of spatio-temporal patterns (e.g. a consistent delay of the mouse behind the gaze or vice versa shown through the level of dynamic interaction between gaze and mouse trajectories) and so could be used in our new analytical methodology. For this, the participants were presented with geometric objects on the screen as a proxy for routes on maps and asked to perform the following three tasks:

- Task 1 –natural tracing. “*Trace the object with the mouse*.”
- Task 2 –eyes-first. “*Trace the object so that your eyes are moving first and the mouse is following the gaze*.”
- Task 3 –mouse-first. “*Trace the object so that your mouse is moving first and the eyes are following the mouse*.”

The tasks were defined to exhibit the expected level of dynamic interaction in each case, which was based on the combination of conscious and subconscious control of the eyes as opposed to the conscious movement of the hand. Hand movements are often (but not always) controlled consciously, while people are often neither aware of their eye movements nor they always consciously control them [23]. However, it *is* possible to voluntarily move the eyes both when executing saccadic motion and smooth pursuit. We defined the three tasks to encompass the three possible combinations of conscious and subconscious eye movements, as per the following:

- Task 1 –natural tracing. This task was devised to observe the natural behaviour: using subconscious saccadic movements of the eye while consciously tracking the geometric shape with the mouse.
- Task 2 –eyes-first. This task was defined to observe a combination of conscious mouse movement and conscious saccadic eye movement. This behaviour was expected to be the most ‘unnatural’, forcing the use of peripheral (or perhaps parafoveal) vision in observing the mouse and performing voluntary saccades to the locations outside of the foveal area, identified with the covert attention. Even though peripheral vision is sensitive to motion, this instruction would force the participant to constantly switch between overt and covert attention while checking the actual location of the mouse.
- Task 3 –mouse-first. This task was devised to observe a combination of conscious mouse movement and conscious eye movement of the ‘smooth pursuit’ type, where the eye was expected to lock onto the pointer that was moving ahead.

In terms of the level of dynamic interaction between the gaze and the mouse, we expected to see the highest level in task 3, i.e., the eye and the mouse would move most closely to each other during the entire task. We further hypothesized that task 2 would be the most difficult one to execute due to the constant switching between overt and covert attention, therefore we expected to see the lowest level of dynamic interaction between the gaze and the mouse. For task 1, we expected that the dynamic interaction would be somewhere in-between tasks 2 and 3, but very likely closer to task 3 than to task 2.

The stimuli (the four geometric shapes) consisted of two primitive shapes (a circle and a rectangle) and two more complex ones with curvy and edgy characteristics (a flower-like curve and a pentagonal star). We varied the geometric shapes in their levels of complexity to control for the effect of complexity in the eye and mouse movements; however, we designed them so that they were consistent in all other aspects, i.e., they were all of the same scale, drawn with a line of the same width and grey level and centred on white background of the size 900x900 pixels, which was placed in the centre of the screen. We decided to use a white background in order to remove any other visual elements that could have distracted the participants and mislead the eye or the mouse movement.

### Participants

The experiment was carried out in a controlled laboratory environment at the University of Zurich. Eleven people (five females, six males, average age 36.8, age range 26–55) participated in the study. All participants were local or visiting academics, staff or postgraduate students with university education (or higher). All but one were non-native English speakers with a high level of understanding of English (the tasks were described in English), while one was a native speaker.

### Apparatus

**Fig 1:**
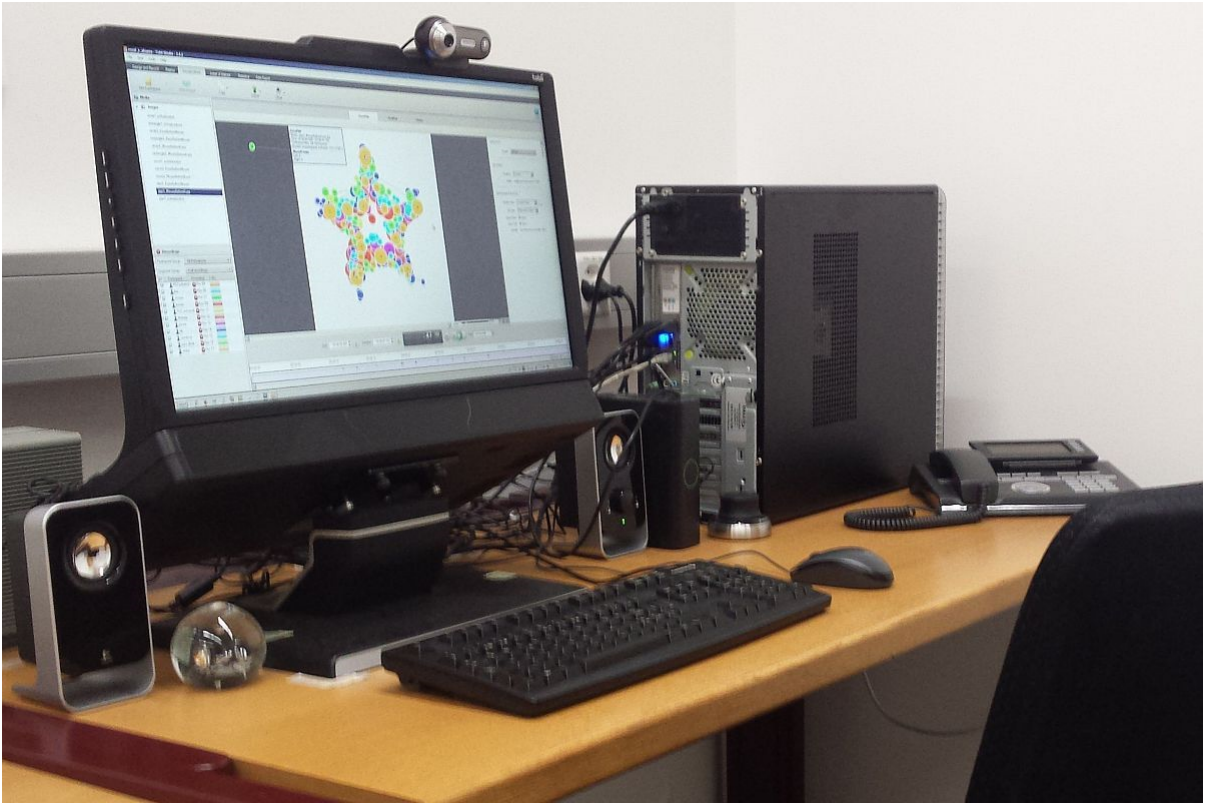
Experimental set up. Windows workstation with a Tobii TX300 eye-tracker in the Geographic Information Visualization and Analysis (GIVA) Eye Movement Lab at the University of Zurich.

The experimental setup was optimized for an eye-tracking study: we used a Windows workstation with a Tobii TX300 eye-tracker, running the Tobii Studio software. Stimuli were displayed on a on a 23-inch flat screen at a 1920*1080 screen resolution, where the stimuli were displayed as 900x900 pixels images, centred on the screen. The participant seat was fixed and located so that the perpendicular distance between the eyes and the screen was 65cm–this distance impacts the visual angle and was thus important in defining the parameters for foveal/parafoveal dynamic interaction measure. Fig 1 shows the experimental setup.

In addition to eye tracking, mouse movements were collected using a free java-based mouse tracking software originally written by Dr Gavin McArdle from University College Dublin (used with permission). The code was adapted by the authors to our particular setup.

### Procedure

Participants were invited through personal contact or email, and scheduled for a session in the lab. When they arrived, they were briefed about the goals of the experiment, i.e. we disclosed the overarching goals but did not tell them our precise goals as we did not want them to try to consciously ‘contribute’ to our goals. They were given the participant information sheet, asked for participation consent and told that if they wish, they can abort the experiment at any time. Once they were seated and eye tracker was calibrated for them, each participant was asked to trace all four geometric shapes using each of the three types of tasks. The four geometric shapes were displayed to each participant in a randomised order to counterbalance for any learning effect, while the three tasks for each shape were always in the same order (1-2-3) not to prime the participants for the task 1 (i.e. the ‘natural tracing’ task always had to appear as the first of the three). At the end of the experiment, the participants could give input/opinion in an open dialogue. After that we thanked them and offered a bar of chocolate. Participants were offered no monetary compensation.

### Data–gaze and mouse trajectories

We collected eye and mouse movement data as they were registered on the screen. Eye movement data consisted of raw gaze trajectories, that is, we used sequences of raw gaze locations where locations were not aggregated into fixations and saccades. Gaze location on the screen was sampled every 3-4ms. These trajectories were captured by and exported from the Tobii Studio software. Mouse movements were captured by sampling mouse position on the screen at 1ms frequency. The coordinate system for both eye and mouse positions was a two-dimensional Cartesian system with the origin point at the upper left corner of the screen, placing the 900x900 pixels stimulus image at the extent of [510,1410] on x axis and [90,990] on y axis. Coordinates of gaze and mouse positions were measured in pixels.

**Fig 2:**
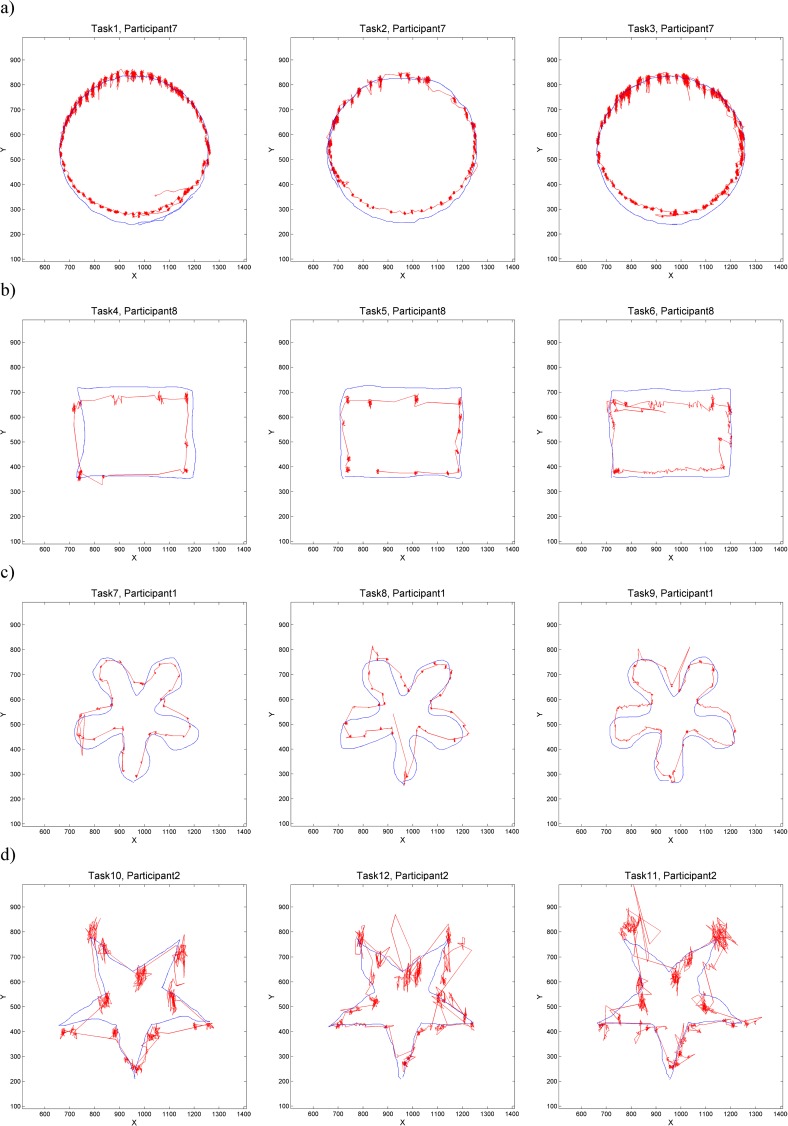
Eye and mouse trajectories for selected participants for all twelve tasks: Natural tracing, eye-first, mouse-first for each geometric shape. Shapes: a) circle b) rectangle, c) curve and d) star. Eye trajectories are shown in red and mouse trajectories in blue.

Gaze and mouse data were matched for each task and participant through time stamps and inspected for spurious starts and ends. That is, if necessary, the starts and ends of trajectories were cut to exclude the eye/mouse movement from before/after the task. For example, after tracing the presented geometric shape, the participant had to move to the next task by pressing a button on the keyboard. For this, almost everyone looked at the keyboard and thus dropped the gaze off the screen: this is part of the gaze trajectory that was still included in the Tobii output (the tracker continued to track the gaze until a specific keyboard button was pressed), but we excluded these spurious tails from our analyses as they are not meaningful for our purposes. Fig 2 shows eye and mouse trajectories for selected participants for all 12 tasks, one participant per form (each row). That is, in each row of Fig 2, we see the gaze and mouse trajectories of the same participant as they were tracing the respective geometric shape with our three tasks (‘natural tracing’, ‘eyes-first’ and ‘mouse-first’). Note how the fixations are clearly observable on the gaze trajectory (the areas with a high concentration of gaze points). There are also noticeable tremors and drift within each fixation and while there are differences in how these look like between participants (e.g. tiny tremors/drifts for participant 1 in Fig 2C and large tremors/drifts for participant 2 in Fig 2D), the spatial pattern of these mini tremors and drifts is consistent for each participant across all three tasks (that is, across the entire row). Note also the difference in geometric properties of the gaze and mouse trajectories for all participants and tasks: the jumpiness of eye-trajectories and the smooth continuity of the mouse trajectories.

**Fig 3:**
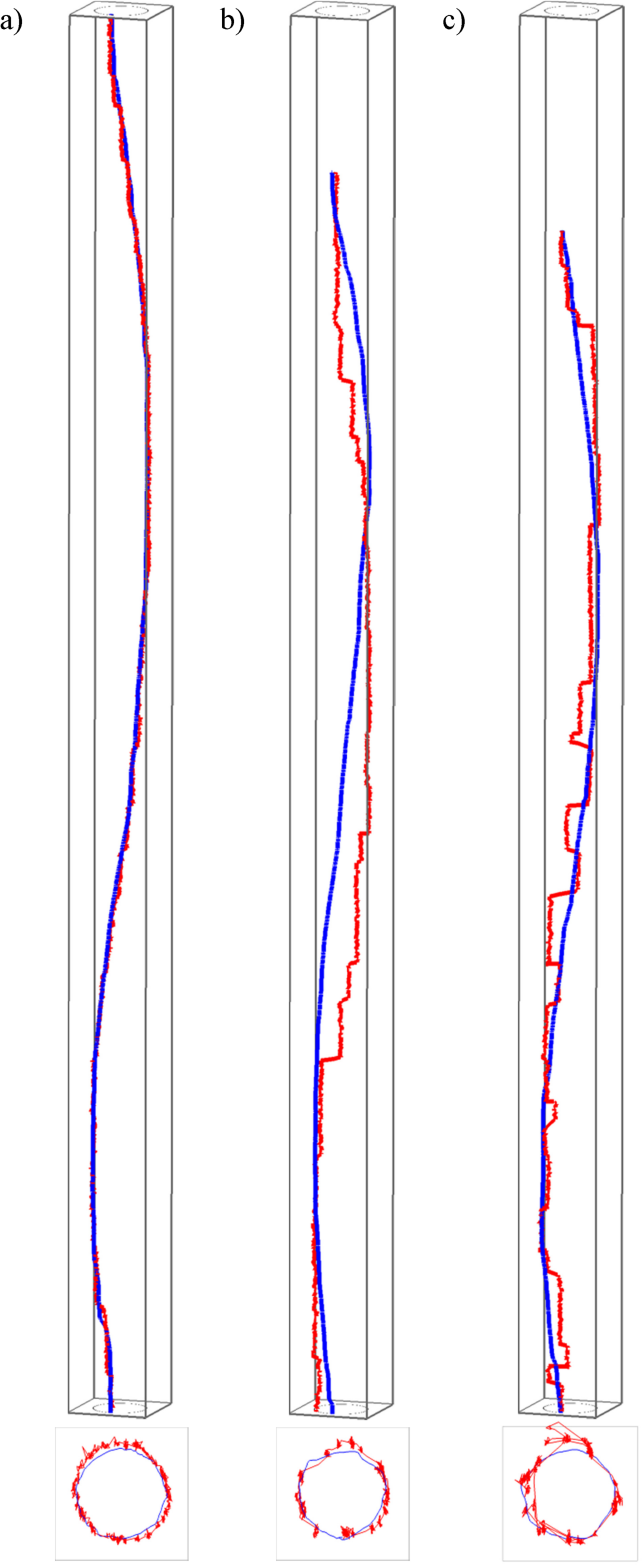
Differences in eye-mouse behaviour across the three tasks. A) task 1, b) task 2, c) task 3. Gaze and mouse trajectories of an example participant (no. 9) are first shown in an STC in red and blue respectively and at the same temporal scale (note the larger differences between eye and mouse for tasks 2 and 3, which require conscious intervention to both hand and eye movement). The lower image in each subpanel is a view from above, that is, a projection of trajectories onto the 2D space of movement.

For this paper we consider that the gaze and mouse are dynamically interacting when the mouse pointer is within the ‘area of attention’ (i.e., where the participant is looking–for more on this, see Analysis Step 3 below, where we link our dynamic interaction measure to the concepts of foveal and parafoveal vision). From Fig 2 it is not possible to say how much dynamic interaction there is at each moment in time nor if there are differences in the level of interaction between participants and tasks. However, if instead of considering eye and mouse trajectories in space only, we consider them in space *and* time, the differences in the interaction levels can be visualised. Fig 3 shows one participant’s trajectories in the STCs, which are displayed at the same spatial and temporal scale for the three tasks shown in this figure. Here, clear differences can be seen between the three tasks–observe how closely the eyes follow the mouse in natural tracing (task 1, Fig 3A), as the two trajectories in the STC differ only very slightly. The ‘eyes-first’ tracing (task 2, Fig 3B) produces the largest differences between the two trajectories and ‘mouse-first’ tracing (task 3, Fig 3C) somewhat smaller differences. Of note are two further observations; that these differences are almost indistinguishable when plotting trajectories only in 2D (see 2D projections at the bottom of the respective STCs in Fig 3), and that surprisingly, already this one example contradicts one of our hypotheses: i.e. that there should be the most interaction and the least difference in the mouse-first task (task 3), where we expected the ‘smooth pursuit’ eye movement to occur. Visual inspection of eye and mouse trajectories from other tasks and other participants confirms this initial observation and we speculate that perhaps it is not fully possible or at least very difficult to consciously perform ‘smooth pursuit’ for an object on the screen (the mouse pointer) that is moved indirectly through the use of another object (the mouse) manipulated by the hand. It is beyond the scope of this paper to further explore this effect, but we note that it could constitute an interesting research question.

Upon inspecting the acquired trajectories, we excluded data from one of the participants from further analysis. This participant consistently had 5% or more of gaze points outside the stimulus area on the screen (the majority of these spurious points were allocated to the coordinates of the origin point, even though the points just before and just after the spurious point were in the same other area of the screen, far from the origin), thus pointing to a potential systemic problem in eye tracker’s detection of this person’s gaze. As one of the goals of this experiment was to produce a data set that could be reliably used to test our new analytical method for quantifying the level of interaction between the gaze and the mouse, we decided to exclude the problematic participant. Our final data set therefore consists of gaze and mouse trajectories of ten participants (four females and six males) on twelve tasks. Anonymised gaze and mouse trajectories for all tasks and participants are provided as Supplementary Information (S1 Data).

## Methodology for dynamic interaction between gaze and mouse movements

In order to develop and evaluate new measures for the level of interaction between gaze and mouse movements, we conducted four separate types of analysis. First we used a traditional HCI evaluation measure, the time spent on task, in order to investigate the level of difficulty. In the second step we investigated the time series of distances between eye and mouse movements. In the third step we propose *a new dynamic interaction measure for gaze and mouse movement*, linked to foveal and parafoveal vision. In the fourth step we compare the measures from steps one and three to see if there is a connection between task times and levels of foveal and parafoveal dynamic interaction.

### Analysis Step 1: Time spent on task

For each task, we calculated time spent on the task as a difference between the last and the first sampling times on the respective gaze trajectory. We calculated means and standard deviations of times for each task across all participants and used a two-way repeated measures ANOVA in order to investigate the effect of the two factors (task type and geometric shape) and their interaction on the mean times (here interaction is meant in ANOVA terms rather than as dynamic interaction). Our expectation was that the tasks that were hypothesised to be the most difficult (task type: eyes-first) would have the longest average task times and that we should see a significant main effect of the task type in ANOVA results. In addition, the shape type could also matter–the two primitive shapes (circle/rectangle) were supposed to be traced faster than the two curved shapes (flower/star), regardless of the task type.

### Analysis Step 2: Time series analysis of gaze-mouse distances

**Fig 4:**
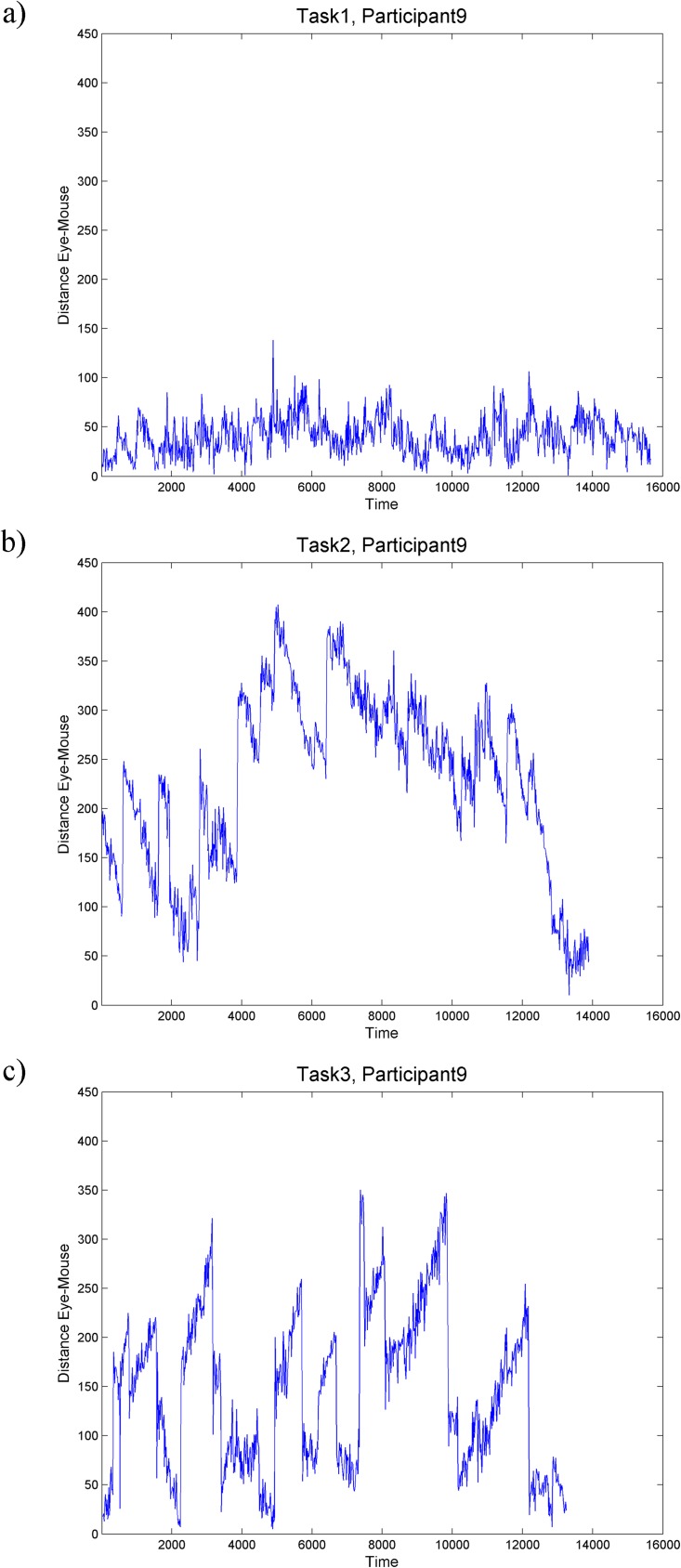
Time series plots of eye-mouse distance. The same participant (no.9) [as in Fig 3] executing the three tasks: a) task 1, b) task 2, c) task 3. For comparison, plots are shown at the same temporal (x axis shows time) and spatial (y axis shows distance) scales.

In this step, we considered gaze and mouse trajectories as time series of locations and calculate the distance between the gaze and the mouse at each moment in time. Fig 4 shows three such distance time series for one participant while executing the three circle-tracing tasks (the same participant and the same tasks as those in Fig 3). We then investigated the temporal distribution of these distances across participants and tasks with the hypothesis that those tasks that are more difficult should have more variation in the gaze-mouse distances and thus the distributions of distance values in each time series should be wider than in series generated in easier tasks.

### Analysis Step 3: Volumetric quantification of dynamic interaction between eye and mouse movements

This section describes our new analytical methodology for quantifying dynamic interaction between gaze and mouse trajectories. We represent movement volumetrically, using space-time densities [24] in order to create what we call a ‘field of influence’ in the STC (see next section for a description). Then we appropriate a common remote sensing change detection method to quantify the extent of interaction in the STC, and define the new dynamic interaction measure as the proportion of interaction voxels in the volume. This methodology is general and could be used for any type of movement. However, because our aim is to work with gaze and mouse trajectories in this project, we link the geometric parameters of the STC volumes to the characteristics of human vision, in particular to foveal and parafoveal vision.

#### Field of influence: A space-time density volume around each trajectory

We define a new measure for quantifying dynamic interactions between the gaze and mouse trajectories based on an overlay of their respective ‘fields of influence’ within the three-dimensional environment of an STC. As mentioned earlier, the physics of eye and mouse movements differ in their basic characteristics, which means that comparing similarity of their trajectories is non-trivial. Mathematically, the comparison should be based on the progression of the two functions (curves) through a 3D space. Each trajectory is a function of time *t* and translates *t* into a location on the 2D screen. One function (mouse trajectory) is continuous and smooth (differentiable) at each *t*, while the other function (gaze trajectory) is, while continuous, not necessarily differentiable at each *t*, since the almost-instantaneous saccades create jump discontinuities in the trajectory. In addition, during each fixation when the eye position should be stationary, the gaze trajectory is jiggled from the exact location of the fixating target through a combination of small irregular tremors and drifts. The STCs in Fig 3 illustrate these two different behaviours.

In computational movement analysis, trajectories are compared based on their physical movement parameters [22], which are often calculated through differentiation on time (e.g. deriving velocity, acceleration and other parameters through numeric differentiation of location coordinates). The non-differentiability of gaze trajectories means that these parameters cannot be calculated during saccades, when the eye is in transition between two fixations and when saccadic suppression occurs. In addition, during a fixation, the irregularity of tremors and drifts could affect the calculation of instantaneous velocity and acceleration and produce artificial outliers that would prevent any direct comparison with the smooth movement of the mouse.

To address this, we propose to represent each movement as a volume, and compare the two volumes instead of directly comparing the shape of the two trajectories. Volumetric representation of movement uses volumes, which are regular 3D grids consisting of voxels (unit cubes, analogous to pixels in a 2D grid). Movement is then represented through values of the probability function calculated using a 2D spatial kernel at each voxel layer and centred on the movement trajectory. The resulting probability value is assigned to the respective voxel based on the distance of the voxel from the trajectory and the resulting volume is called the *space-time density* [24].

**Fig 5:**
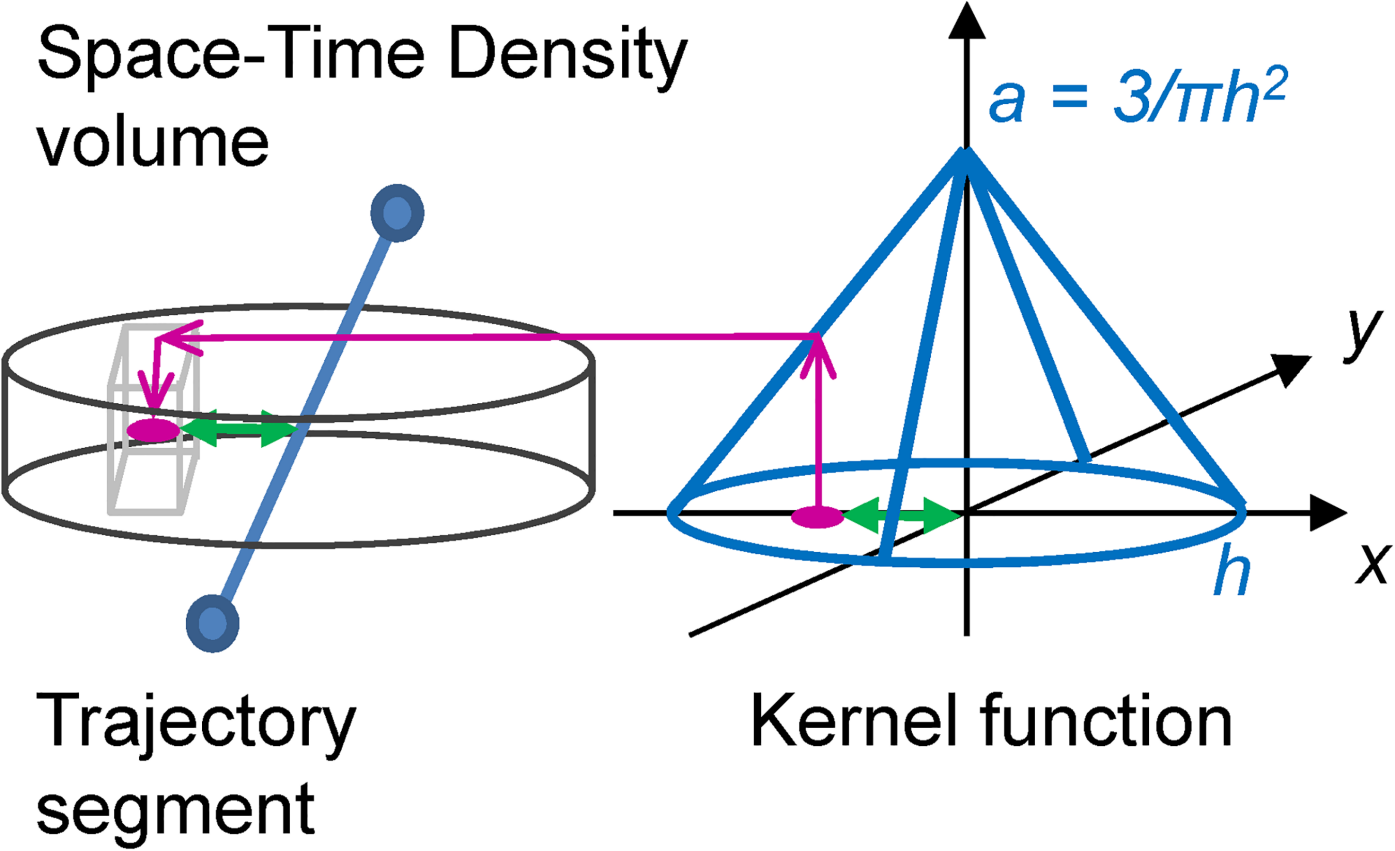
Building the space-time density volume from distance kernels around trajectory. At each voxel layer, the algorithm finds the distance between each voxel (shown as a light-grey cube on the left) and the trajectory (in blue, distance shown with a green arrow), then assigns the value obtained from the kernel function at this particular distance in the kernel space (represented by the 3D coordinate system on the right) to the respective voxel. We link the kernel size (given by the radius h–the bandwidth) to the theory of foveal and parafoveal attention. We further assume that if a person is looking at a certain point, the attention away from the point will decrease linearly with distance and will be cut off at distance h, where h marks the boundary for foveal or parafoveal attention. We therefore use a linear kernel function in the form of a cone in kernel space.

In the context of gaze trajectories, this method of representing movement solves both problems with saccadic jumps, and with irregular micro movements during fixations, as both these types of irregularities are smoothed with the application of the probability function. We therefore calculate space-time densities for each separate trajectory of the gaze and the mouse. These density volumes consist of stacks of two-dimensional kernels, one kernel at each temporal voxel layer (Fig 5). Each kernel is a 2D probability density function (PDF), centred on the gaze or mouse position at a particular moment in time. Similar PDFs have been recently used in eye-tracking to determine the probability of the user attending a certain object in a video game at a particular time [61]. In our case, as the layers of kernels are stacked one upon another through time, this builds a volumetric representation of movement which fills the entire STC. This density represents what we call a ‘*field of influence*’ around a trajectory and the task of comparing the two movement trajectories now becomes the task of comparing the two fields of influence. This is mathematically much simpler than comparing gaze and mouse trajectories directly, since the new comparison is between two volumes, and can be done voxel-wise.

We compare the two volumes using a change detection method generalised into the STC from remote sensing [25]. One of the most commonly used change detection methods in optical remote sensing is image differencing [62]. In this method, two satellite images of the same area and of the same spatial resolution (pixel size) that are taken at two different times, are subtracted, pixel-by-pixel in order to build a difference image. The pixels of the difference image are classified into two groups, those that represent change, and those that represent no change, based on a pre-defined decision function which is set based on the characteristics of the phenomenon under observation. The result is a map of areas where change occurred between the two times when images were taken. We apply a similar principle to our two density volumes, generalising the image differencing idea into three dimensions and adapting the decision function to the dynamic interaction of gaze and mouse.

**Fig 6:**
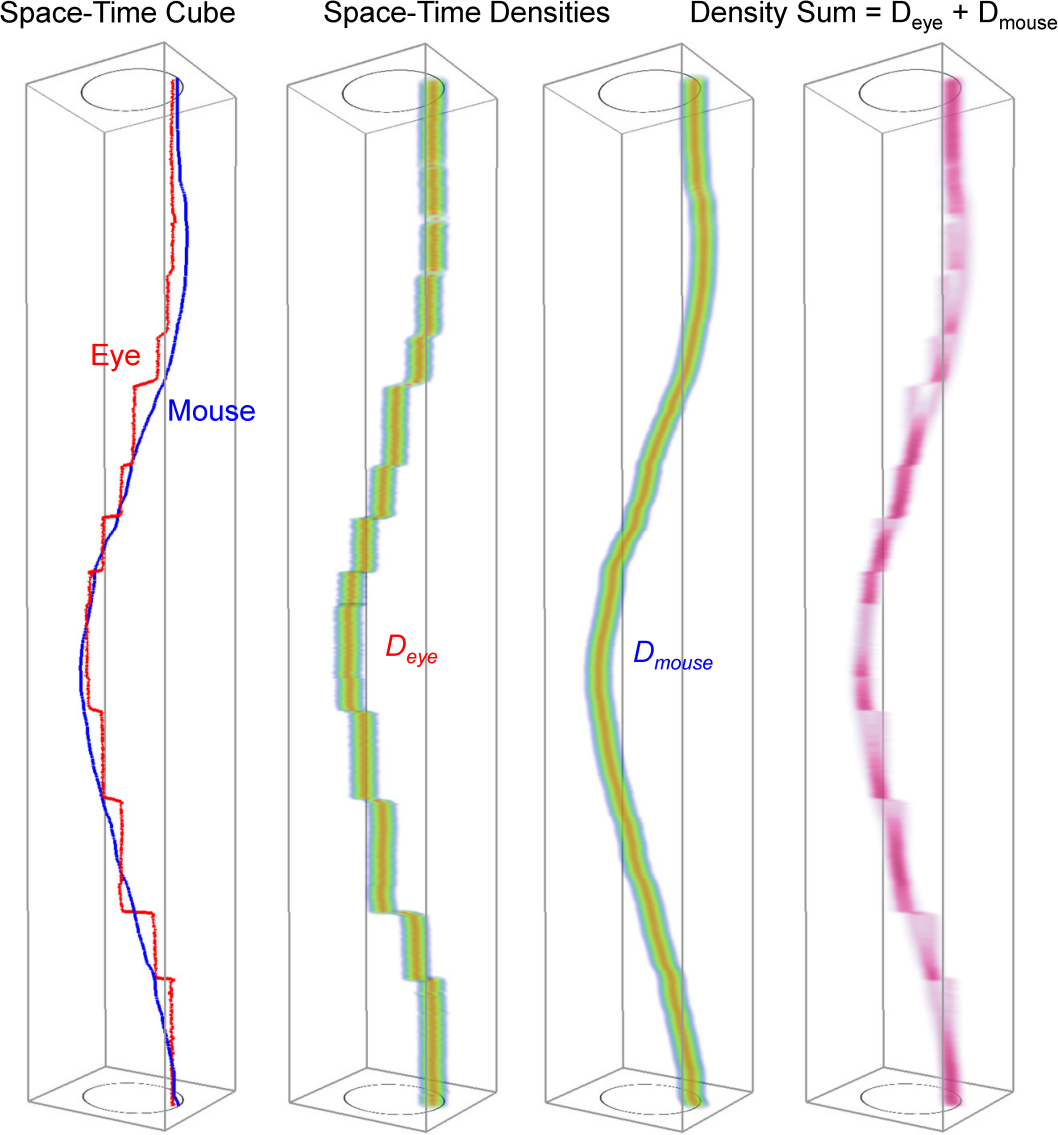
Our proposed analytical methodology to quantify the level of interaction. From trajectories, through densities, to density sum volume in which interaction voxels are counted.

**Fig 7:**
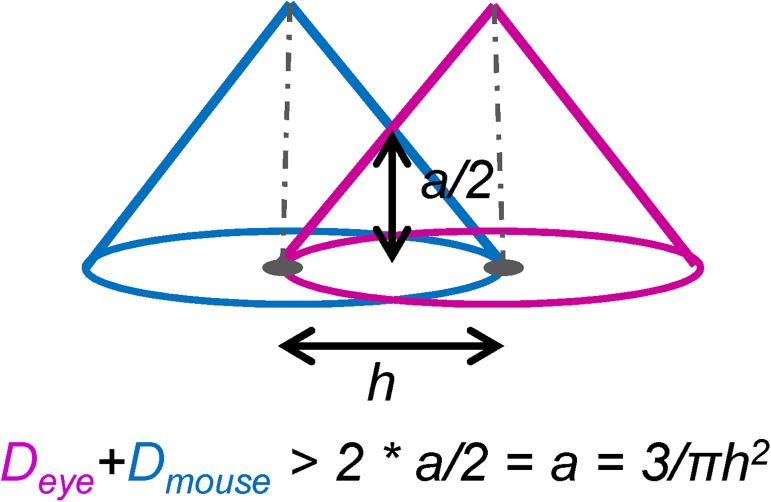
Definition of the dynamic interaction measure from the intersection of density volumes. We define that dynamic interaction occurs in voxels where the two kernel centres are less than *h* apart from each other. In these voxels, values of the density sum are larger than twice the height at this distance from the centre, which is half of the total height of the linear kernel (*a/2*, see also Fig 5).

We calculate a voxel-by-voxel sum of gaze and mouse densities (Fig 6). The reason for choosing the sum as our algebraic operation rather than the original difference is that we are interested in areas in space and time when gaze and mouse are within each other’s field of influence. Fig 7 illustrates this idea: imagine the position of the gaze as the centre of the pink kernel and the position of the mouse as the centre of the blue kernel. The width of the kernel, the bandwidth, is marked with h and the height of the kernel is equal to *a = 3/πh*^*2*^ (see also Fig 5) for the linear kernel to be a 2D PDF (which means that the volume under the kernel has to integrate to 1). We mathematically define the dynamic interaction to occur when the gaze and the mouse are closer to each other than the bandwidth *h* (we will further link *h* to the concept of foveal/parafoveal attention). From this, we can calculate that for the dynamic interaction to occur, the two linear kernels need to intersect at the height of *a/2* each, that is, the sum of the two kernels needs to be larger than 2*a/2 or *a* (Fig 7). This is the threshold to be used as a decision function to classify voxels into interaction voxels vs. non-interaction voxels. In other words, if a voxel of the density sum volume has the value larger or equal to *a*, we count it as an interaction voxel.

The last step in the definition of the dynamic interaction measure is the normalisation of the count of interaction voxels with the total number of voxels in the volume. This is done in order to obtain a comparable measure of dynamic interaction for different cases and different volume sizes (note that volume sizes are dependent only on the time spent on task, since bases of all STCs are of the same size, i.e. the image stimuli from our experiment).

#### Method comparison

**Fig 8:**
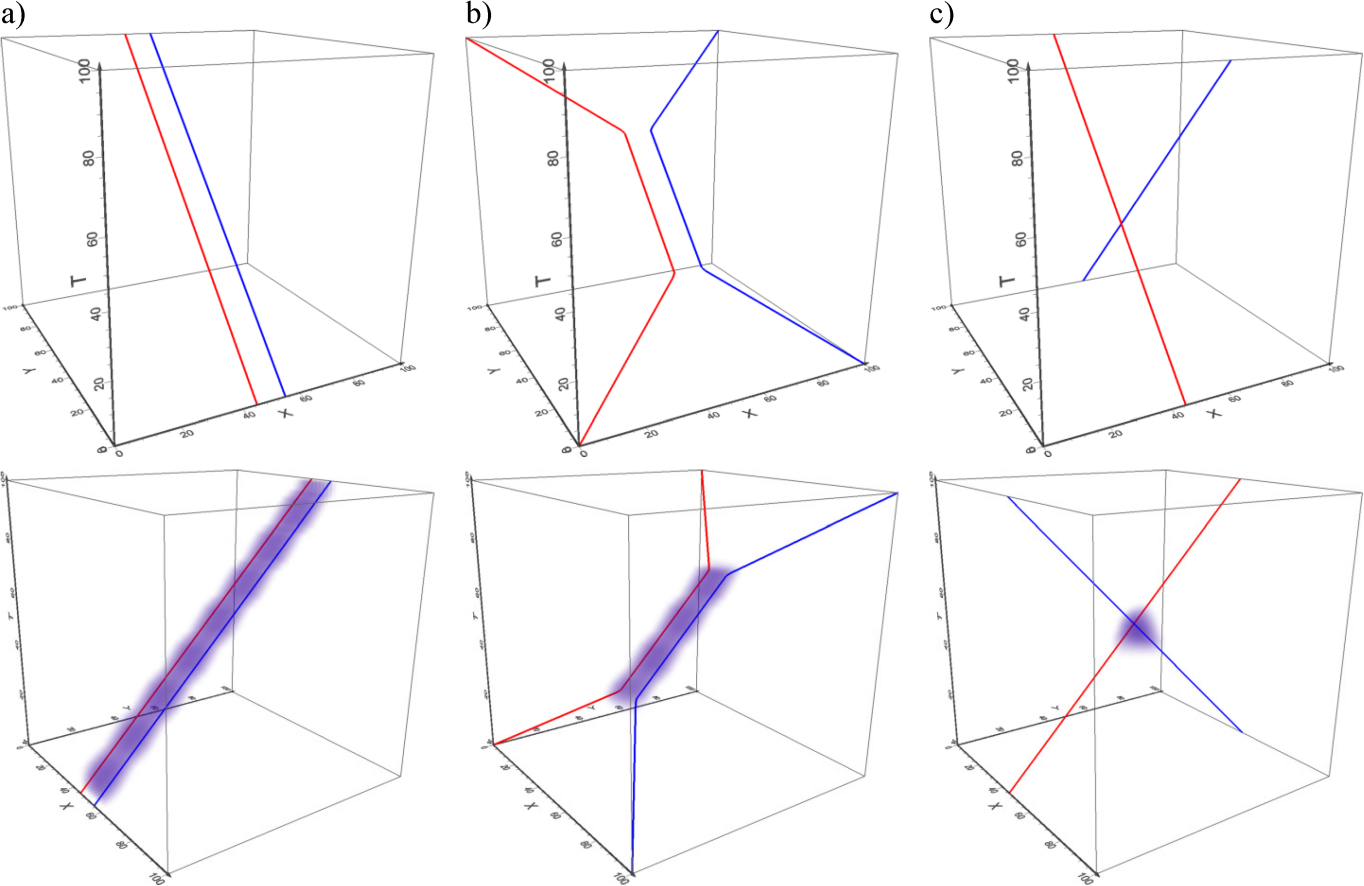
Simulated data for method comparison with a) full dynamic interaction (case 1—trajectories parallel in space and time), b) middle level dynamic interaction (case 2) and c) almost no dynamic interaction (case 3—trajectories parallel in space but not time). Bottom panels show our interaction volumes for each case (voxel size 2, kernel size 20).

Our method is as far as we know the first method that attempts to quantify the dynamic interaction between two physically diverse movement types. However, a number of methods exist for dynamic interaction between similar movement types, in particular in movement ecology. To evaluate our method we therefore compare our dynamic interaction measure with two ecological measures [41] and as the latter two are not suitable for eye and mouse data, we do so on simulated trajectories. We created three cases with simulated data, each of which has a different levels of dynamic interaction (Fig 8). Simulated data are provided as Supplementary Information (S2 Data).

The two ecological indices we are using for comparison are Prox and HAI [41]. Prox is a proportion of the spatio-temporally proximate fixes in two given trajectories among all the temporally proximate fixes. The range of this index is [0,1], where values near 1 indicate a high level of dynamic interaction. HAI is similar, except the spatio-temporally proximate fixes are not counted across the entire area, but only within the so-called overlap zone, which is the intersection of the home ranges of the two animals. Home range is a term from ecology, defined as the set of areas where an individual animal spends most of its time and there are a number of methods how this can be calculated from trajectory data [21]. For our three simulated cases we created the overlap zone for HAI as intersection of buffers around the two trajectories. We calculated the Prox and HAI values for all three simulated cases and compared them with our dynamic measure.

#### Sensitivity analysis

Our method uses the volumetric representation of movement and is therefore dependent on the resolution of the spatio-temporal partitioning of data space, that is the size of the voxels in the volume. Another parameter that can be adjusted is the kernel size, which tells us how far from each trajectory does the field of influence reach. We investigated how the voxel size and the kernel size affect results by running two experiments on the same simulated data as above, where we kept one of the two parameters constant and changed the second parameter.

#### Linking interaction volume parameters to properties of human vision

The previous section described the mathematical definition of our new measure for dynamic interaction and the process of its derivation. So far, the derivation was general and could be used for comparing any two types of movement, however, as we are working with eye movement data we wanted to link it to the specifics of human vision. For this, we link the parameters of the space-time density to the concepts of foveal and parafoveal attention.

**Fig 9:**
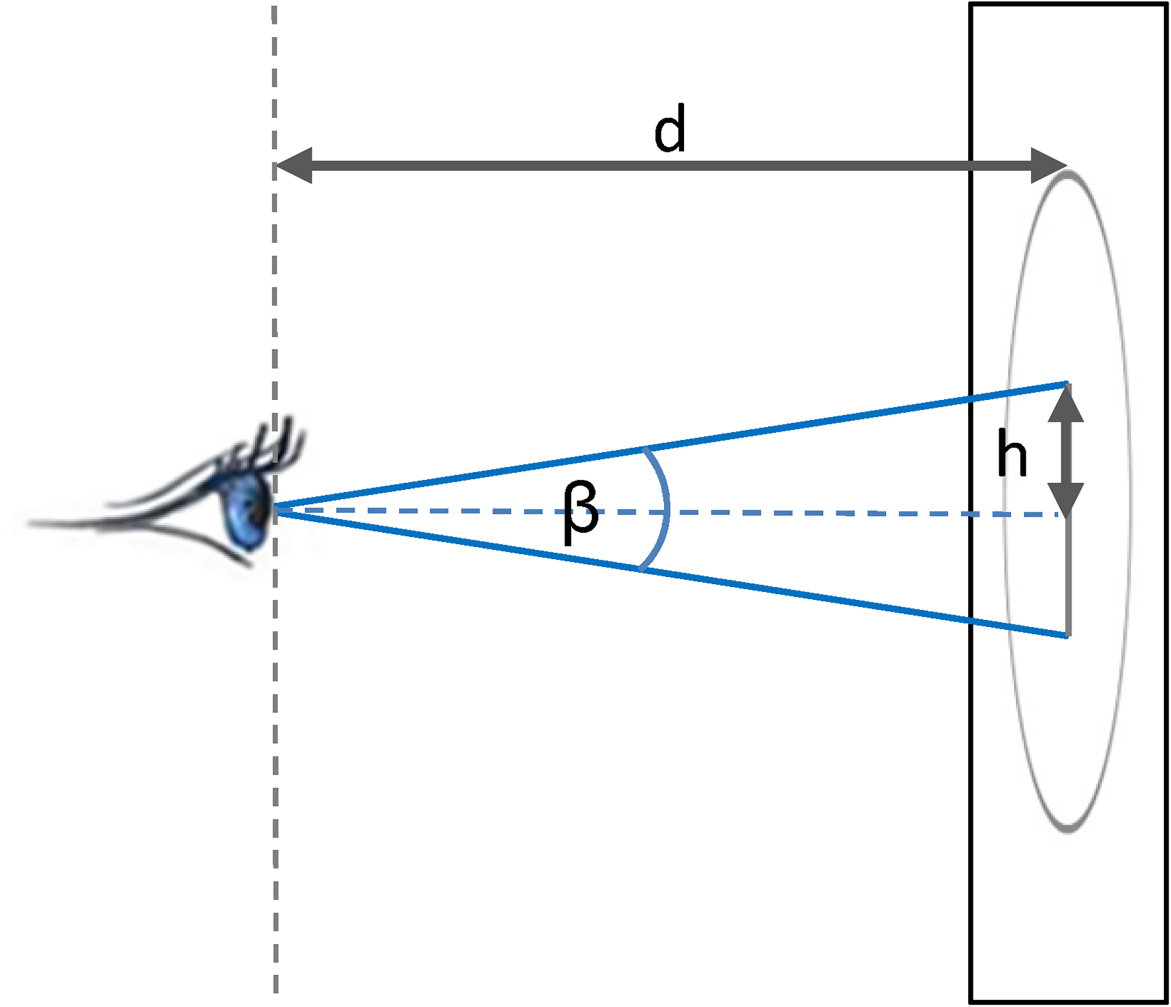
Linking density volume parameters to characteristics of human vision. Here, *d* is the fixed distance from the participant to the screen (in our case 65cm), while *h* that we use as the kernel size for the density volume (see Fig 5), depends on the view angle *β* (or rather, on one half of this angle, *β /2*). *β* is set to 2° for foveal interaction to 5° for parafoveal interaction.

As described previously, the human visual field is spatially divided into three regions: foveal, parafoveal and peripheral, based on the angle *β* formed around the line of vision (Fig 9). Foveal region corresponds to the visual angle of 2°, parafoveal region to the angle of 5° and the periphery corresponds to anything beyond the 5° [26,28]. We use the geometrical definitions of the foveal and parafoveal regions to set the values for the bandwidth *h* of our space-time densities. Given the distance *d* of the eyes from the screen and the size of the visual angle *β*, we can calculate the radius *h* of the region that this particular visual angle covers:

h=d*tan(β/2) (1)

*β/2* is the angle between the visual axis, where the fovea is aligned to and the location away from the fovea and is also called the angle of eccentricity [30, 27]. Given the parameters of our geometric setup we can calculate the *h* for foveal and parafoveal regions and use these two values to set the bandwidth for space-time densities for foveal/parafoveal interaction. This means that our dynamic interaction measure as defined above will identify the proportion of voxels where the mouse is closer to the eye than *h* (as explained above and in Fig 7), that is, within the foveal/parafoveal region respectively. This is similar to the 2D approach of Sundstedt et al. [63] who model the 2D energy spread of the gaze by placing a kernel on the location of the fixation (kernel is defined by the size of the foveal region) and use this kernel to link gaze to the geometric shape of the underlying object. In our case, the energy spread is modelled by the gaze kernel and the underlying object is the mouse, which is modelled through a separate kernel as explained above. Table 1 provides values of geometric parameters calculated from the setup measures and used for derivation of the parameters for the two sets of space-time densities.

**Table 1:** Geometric parameters for calculation of gaze and mouse space-time densities.

Geometric parameter	Value
Stimulus size	900 x 900 pixels
Pixel size*	0.265mm
Voxel size in *x/y* directions	10 pixels
Voxel size in *t* direction	10ms
Perpendicular distance *d* from eye to screen	65cm
Radius of foveal region (*β* = 2°), calculated as per Eq (1)	1.135cm = 43 pixels
Kernel size h for density foveal interaction calculation**	50 pixels
Radius of parafoveal region (*β* = 5°), calculated as per Eq (1)	2.838cm = 107 pixels
Kernel size h for density in parafoveal interaction calculation**	110 pixels

* Pixel size was calculated as the ratio between screen height (cm) and height of the stimulus image (pixels).

** Radius rounded to the first next multiple of voxel size in x/y directions.

#### Differences in levels of foveal and parafoveal dynamic interaction

Once we obtained the dynamic interaction measures for all participants in all tasks in this way, we investigated if there were any differences in the levels of foveal or parafoveal dynamic interaction for task types and geometric shapes. We ran two-way repeated measure ANOVAs to investigate the effect of the two factors or their statistical interaction on task time and the levels of foveal and parafoveal dynamic interactions. For this, we performed two two-way repeated measures ANOVAs with task type and geometric shape as the two factors, and respectively with foveal or parafoveal dynamic interaction as the dependent variable.

### Analysis Step 4: Linking time spent on task to the level of dynamic interaction

In the final step of the analysis, we investigated if the time spent on task was related to the levels of foveal and parafoveal dynamic interaction. For this, we calculated correlation coefficients between time and both dynamic interaction levels respectively for each task.

### Software and code

The analysis was coded in Matlab and R and the 3D figures produced in Voxler software for volumetric visualisation. R code that implements our new analytical methodology (space-time densities and calculation of dynamic interaction between two trajectories) is provided as Supplementary Information (S1 Code). We used the R-package wildlifeDI for calculation of the two ecological indices Prox and HAI [41].

## Results

### Analysis Step 1: Time spent on task

**Fig 10:**
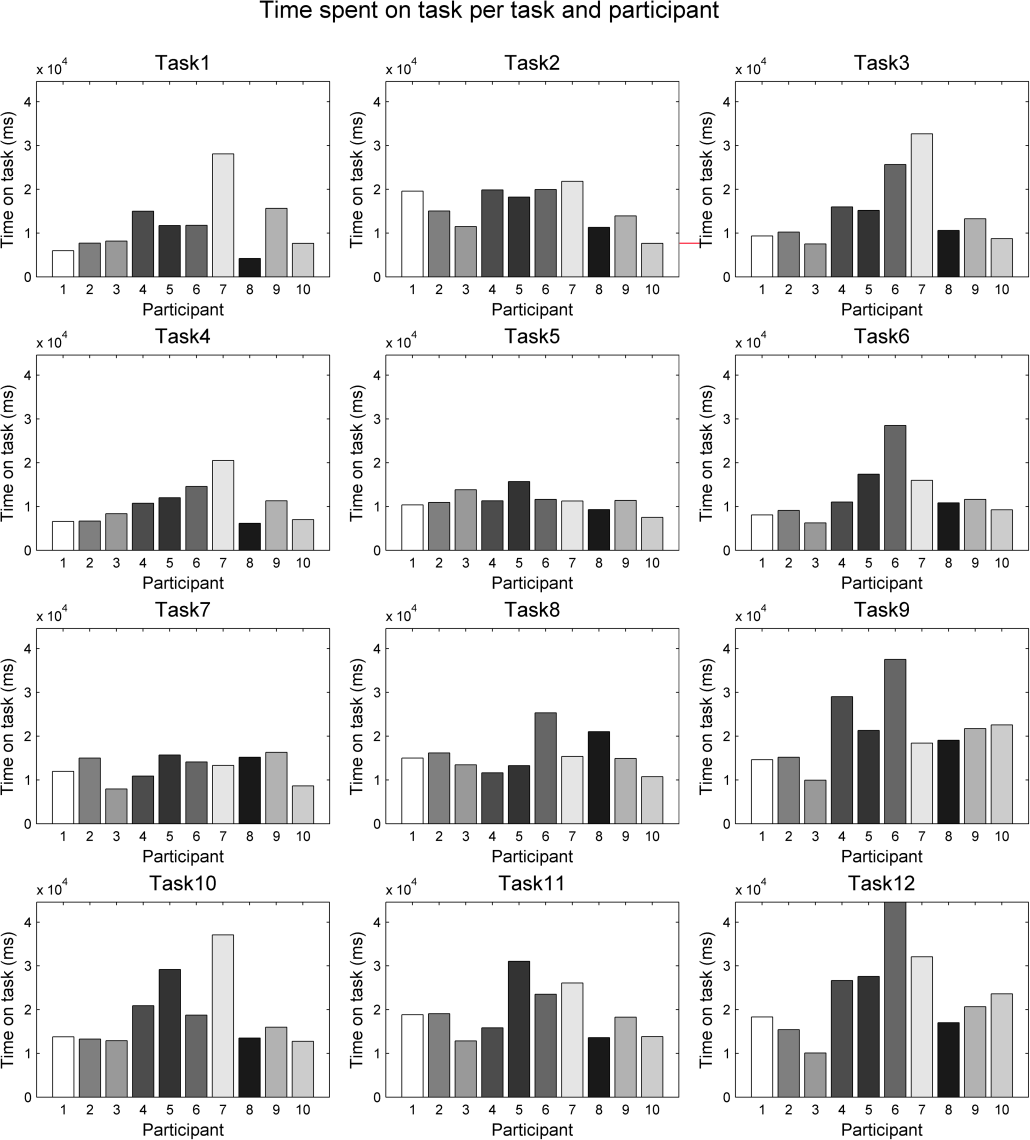
Times spent on task. Times are shown on the same scale per task and participant.

Fig 10 shows bar charts of times spent on task per task and participant, shown at the same scale. Mean times and standard deviations for each task are shown in Table 2.

**Table 2:** Mean and standard deviation of times spent on task. Note that the task numbering here (1–12) is for convenience of reporting, and it does not express the task *types* as introduced in the experiment design section. To make the link, the ‘tracing method’ column is important (i.e., experimental tasks are repeated based on tracing method per shape type).

Task No.	Shape	(Task type #) Tracing method	Time on task (ms):	
			Mean	St Dev
1	Circle	(1) Natural	11583	6908.4
2	Circle	(2) Eyes-first	15868	4713.0
3	Circle	(3) Mouse-first	14902	8165.3
4	Rectangle	(1) Natural	10385	4543.1
5	Rectangle	(2) Eyes-first	11310	2242.6
6	Rectangle	(3) Mouse-first	12788	6475.5
7	Curve	(1) Natural	12888	2945.1
8	Curve	(2) Eyes-first	15675	4412.1
9	Curve	(3) Mouse-first	20926	7810.8
10	Star	(1) Natural	18808	8228.3
11	Star	(2) Eyes-first	19290	5956.2
12	Star	(3) Mouse-first	23606	9815.1

Interestingly, we do not see the expected increase in task times for tasks with the eyes-first tracing method (the middle column of Fig 9). However, unsurprisingly, we see an increase in the time-use across geometric shapes, with the two simpler shapes (the rectangle and the circle) that are composed of shorter lines and fewer turns having the shortest completion times, followed by the curve and the star. In particular, the star stands out as the shape requiring the longest times. Across tracing methods, there is more variation, but on the whole, it was the third type of tracing (mouse-first, the right column of Fig 9) that people used the longest time to perform.

**Fig 11:**
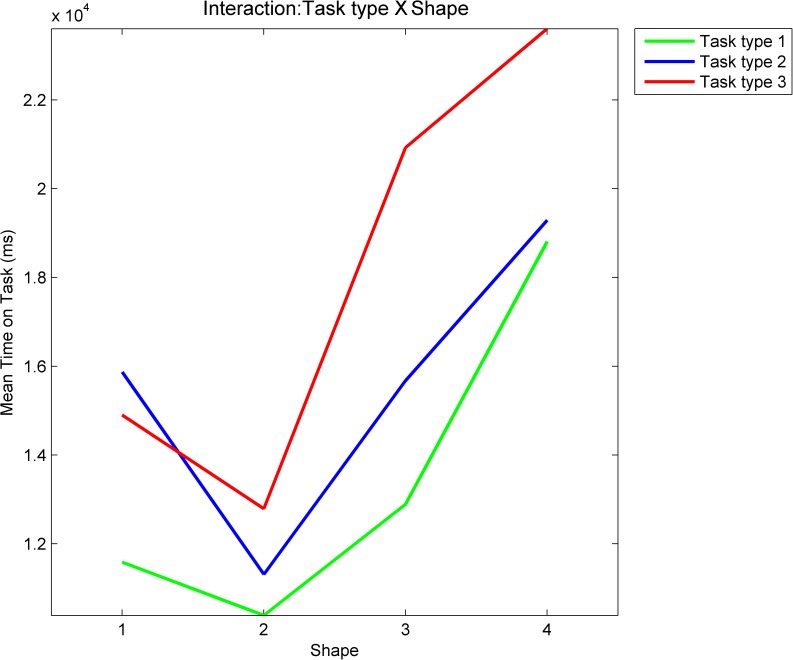
ANOVA plot for time spent on task. The plot shows ANOVA interaction between task type and geometric shape. Task types are: 1 –natural tracing, 2 –eyes-first and 3 –mouse-first. Shapes are: 1 –circle, 2 –rectangle, 3 –flower and 4 –star.

The two-way repeated measures ANOVA on task time confirmed observations from the previous paragraph. There is a significant main effect of task type (F = 4.1555, p = 0.0328<0.05), there is a highly significant main effect of geometric shape (F = 14.0760, p = 6.1837*10^−6^<0.001) and there is a significant effect of statistical interaction between the two factors (F = 3.4133, p = 0.0063<0.05). Fig 11 shows the ANOVA plot. Interestingly (and somewhat contradictory to our expectations), the longest mean times are for task type 3 (mouse-first), rather than task type 2 (eyes-first), which we hypothesised would be the most difficult task to execute. Fig 11 also shows that the ANOVA interaction is between these two task types, while the task type 1 (natural tracing, shown with a green line in Fig 11) is separate with the shortest mean times for all four geometric shapes.

### Analysis Step 2. Time series analysis of eye-mouse distance

**Fig 12:**
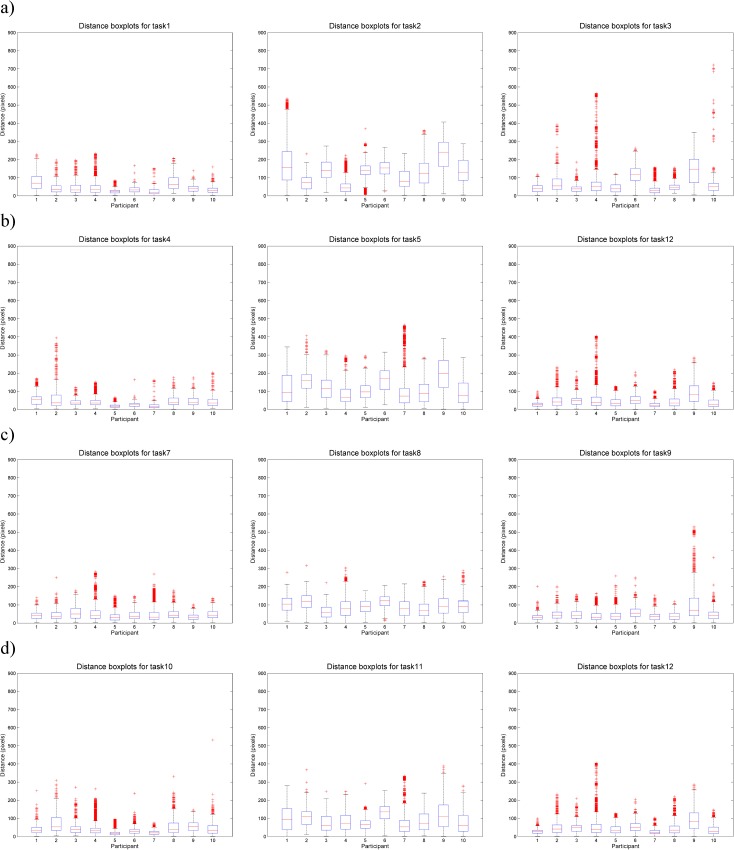
Box-and-whiskers plots of gaze-mouse distance per participant. Plots are shown for tracing a) the circle, b) the rectangle, c) the curve and d) the star. Each row shows three charts: for natural tracing, for eye-first and for mouse-first. Each boxplot in each chart is the distribution of eye-mouse distance for one participant, where participants are ordered in the same way across all charts and all charts are shown at the same scale, to allow for visual comparison across each geometric form (per row) and across all forms (per column).

Fig 12 shows the box-and-whiskers plots of variations of eye-mouse distances. That is, for each eye-mouse distance time series we take all distance values and produce one plot per task and participant. All plots are shown at the same scale and all participants in the same order, to make subpanels of this Fig visually comparable with each other. Each box is crossed by the red median line and defined by the 25^th^ and 75^th^ percentiles. The whiskers extend to 1.5 intra-quartile differences on each side and all points beyond that are individually plotted as outliers (in red).

The distribution of distance values in each time series can be investigated looking at the sizes of the boxes and the length of the whiskers. In general, natural tracing tasks (the left column of Fig 12) have the smallest boxes, the eye-first tasks (the central column of Fig 12) have the largest boxes with only a few outliers, while the mouse-first tasks show more individual variation (e.g. participant no. 9 has consistently a much larger box in the mouse-first tasks than any other participant, see the third column of Fig 12). This seems to indicate that when naturally tracing a shape, the gaze and mouse are consistently closer at each moment in time than when tracing with eyes-first–a result that confirms our hypothesis that there should be the least interaction between gaze and mouse in eyes-first tasks. The results for mouse-first are less clear (the right column of Fig 12)–while boxes in the respective plots are similarly narrow as in natural tracing tasks, there are substantially larger outliers present for several individuals, including one individual (no. 9) whose distribution of distance values for mouse-first tasks is similar to his/her pattern for eyes-first task rather than natural tracing tasks.

### Analysis Step 3: Quantifying the dynamic interaction

#### Method comparison

The Prox and HAI indices show the expected pattern in the interaction level for our three cases: case 1 –high interaction, case 2 –mid-level interaction and case 3—low interaction. Our measure (DI Eye-Mouse) shows the same decreasing pattern (Table 3). Note that Prox and HAI values are the same for cases 1 and 3, due to the definition of the overlap zone, which for these simulated data means that in both cases all trajectory fixes are counted for both indices. Note also that while Prox and HAI have a fixed range from [0,1], this range depends on the concept of home range, which is a spatial concept and does not take into consideration time. Our measure in contrast normalises the interaction using the spatio-temporal volume, meaning that the values are significantly lower in comparison with the ecological indices because of the inclusion of time into the normalisation.

**Table 3:** Values of dynamic interaction indices for three simulated data cases.

Case	Prox	HAI	DI Eye-Mouse
1	1	1	0.02371
2	0.445545	0.714286	0.01079
3	0.168317	0.168317	0.00355

#### Sensitivity analysis

**Fig 13:**
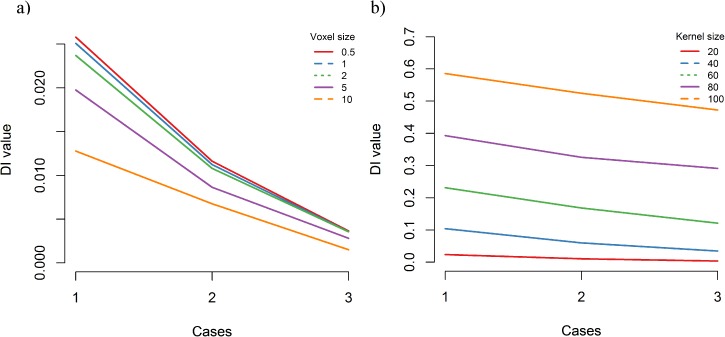
Sensitivity to method parameters shown on simulated data. Panel a) shows changes in method results for varying voxel sizes while kernel size is kept constant. Panel b) varies kernel size for a constant voxel size. The pattern of interaction value decreasing from case 1 through 2 to 3 is the same, regardless of voxel or kernel size.

Table 4 shows the results of both experiments, where in the first one we kept the kernel size constant and varied voxel size and in the second one did the opposite. Fig 13 shows these values broken down by voxel/kernel size in each respective experiment. The pattern where interaction decreases from case 1 to case 3 is the same in all cases, regardless of voxel or kernel variation. Further, the values of the measure increase with kernel size and decrease with voxel size, both of which are expected patterns. These are typical behaviours of algorithms on discretised space representations, such as for example raster algorithms—in our case, we have generalised them into the volumetric space.

**Table 4:** Values of our dynamic interaction measure vs. voxel size and vs. kernel size. In the first two columns we varied the voxel size while keeping the kernel size constant (20). The last two columns show results where kernel size was varied at a constant voxel size (2).

	Experiment 1		Experiment 2	
	Constant kernel size = 20		Constant voxel size = 2	
Case	Voxel size	DI Eye-Mouse	Kernel size	DI Eye-Mouse
1	0.5	0.0258	20	0.02371
1	1	0.02508	40	0.1044
1	2	0.02371	60	0.23121
1	5	0.01976	80	0.39322
1	10	0.01277	100	0.58573
2	0.5	0.01161	20	0.0108
2	1	0.01123	40	0.06016
2	2	0.0108	60	0.16824
2	5	0.00864	80	0.32589
2	10	0.00676	100	0.52475
3	0.5	0.00365	20	0.00355
3	1	0.00356	40	0.03469
3	2	0.00355	60	0.12107
3	5	0.00281	80	0.2911
3	10	0.0015	100	0.57259

#### Quantifying foveal/parafoveal interaction

**Fig 14:**
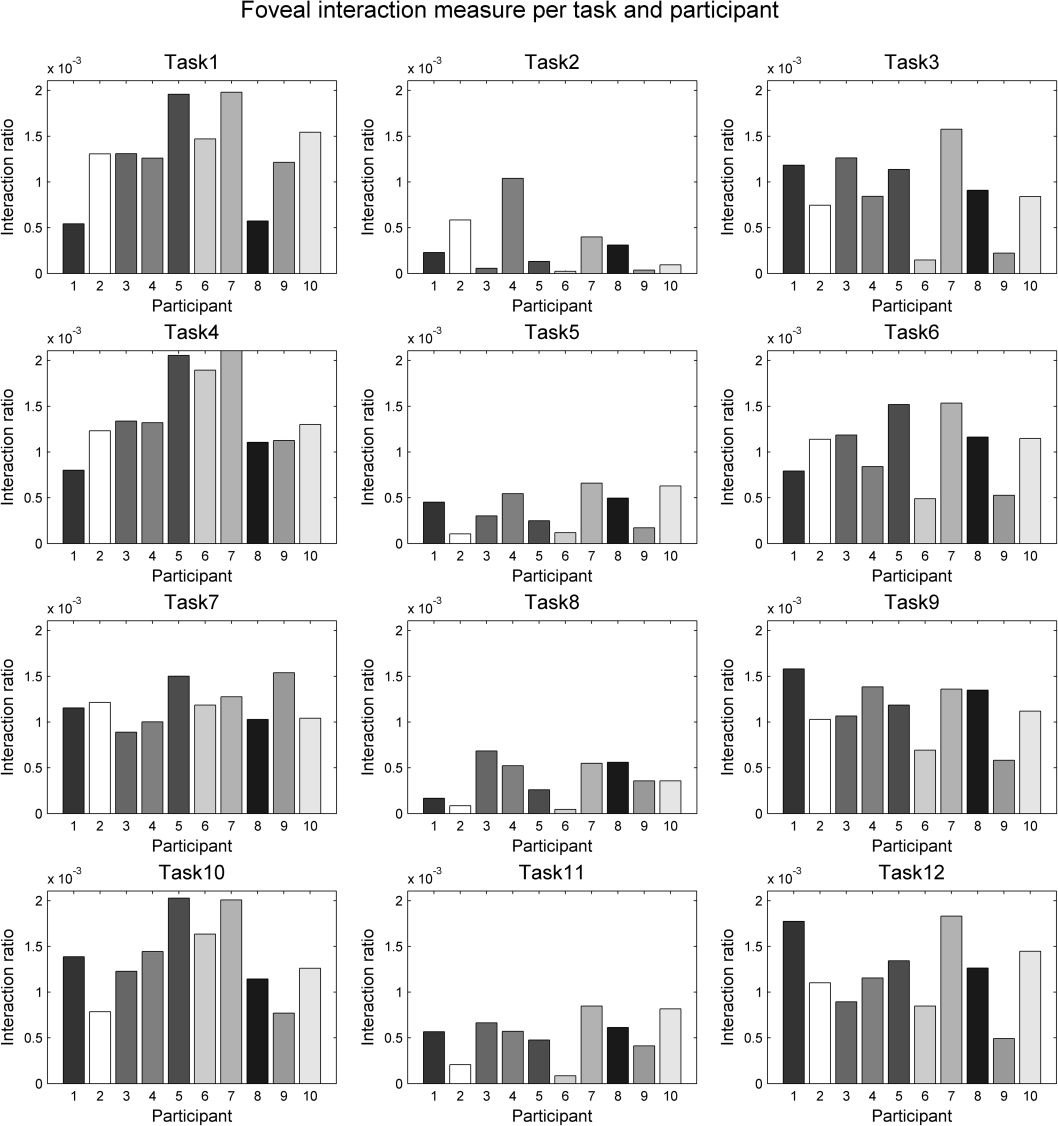
Quantifying foveal interaction. Plots are shown for tracing the circle (tasks 1–3), the rectangle (tasks 4–6), the curve (tasks 7–9) and the star (tasks 10–12). Each row shows three charts: for natural tracing, for eye-first and for mouse-first. Participants are ordered in the same way across all charts and all charts are shown at the same scale, to allow for visual comparison of charts across each geometric form (per row) and across each participant (per each bar in each chart). Bars are coloured per participant in this and the next figure (parafoveal interaction).

**Fig 15:**
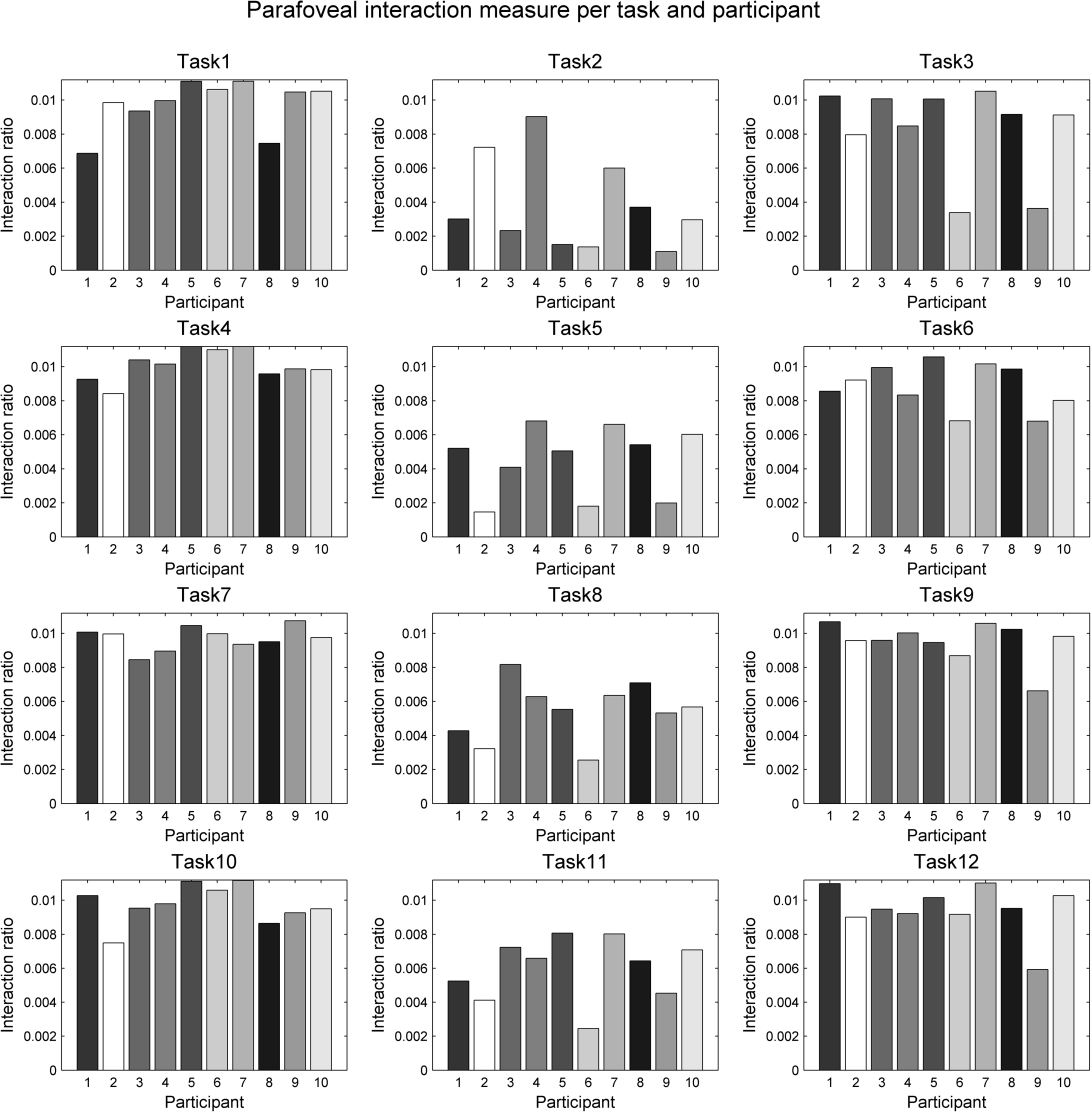
Quantifying parafoveal interaction. Plots are shown for tracing the circle (tasks 1–3), the rectangle (tasks 4–6), the curve (tasks 7–9) and the star (tasks 10–12). Each row shows three charts: for natural tracing, for eye-first and for mouse-first. Participants are ordered in the same way across all charts and all charts are shown at the same scale, to allow for visual comparison of charts across each geometric form (per row) and across each participant (per each bar in each chart). Bars are coloured per participant in this and the previous figure (foveal interaction).

To analyse the level of dynamic interaction between the gaze and the mouse within foveal/parafoveal regions, we plotted our dynamic interaction ratios as bar charts per task and per participant (Figs 14 and 15). Participants are ordered in the same way in all plots in these two Figs and each participant is represented with a consistent shade of grey of respective bars in order to allow for visual comparison of the same person’s performance in all tasks and at both foveal and parafoveal levels. All plots are also scaled the same for foveal interaction and for parafoveal interaction respectively, allowing visual comparison of subpanels within each figure.

**Fig 16:**
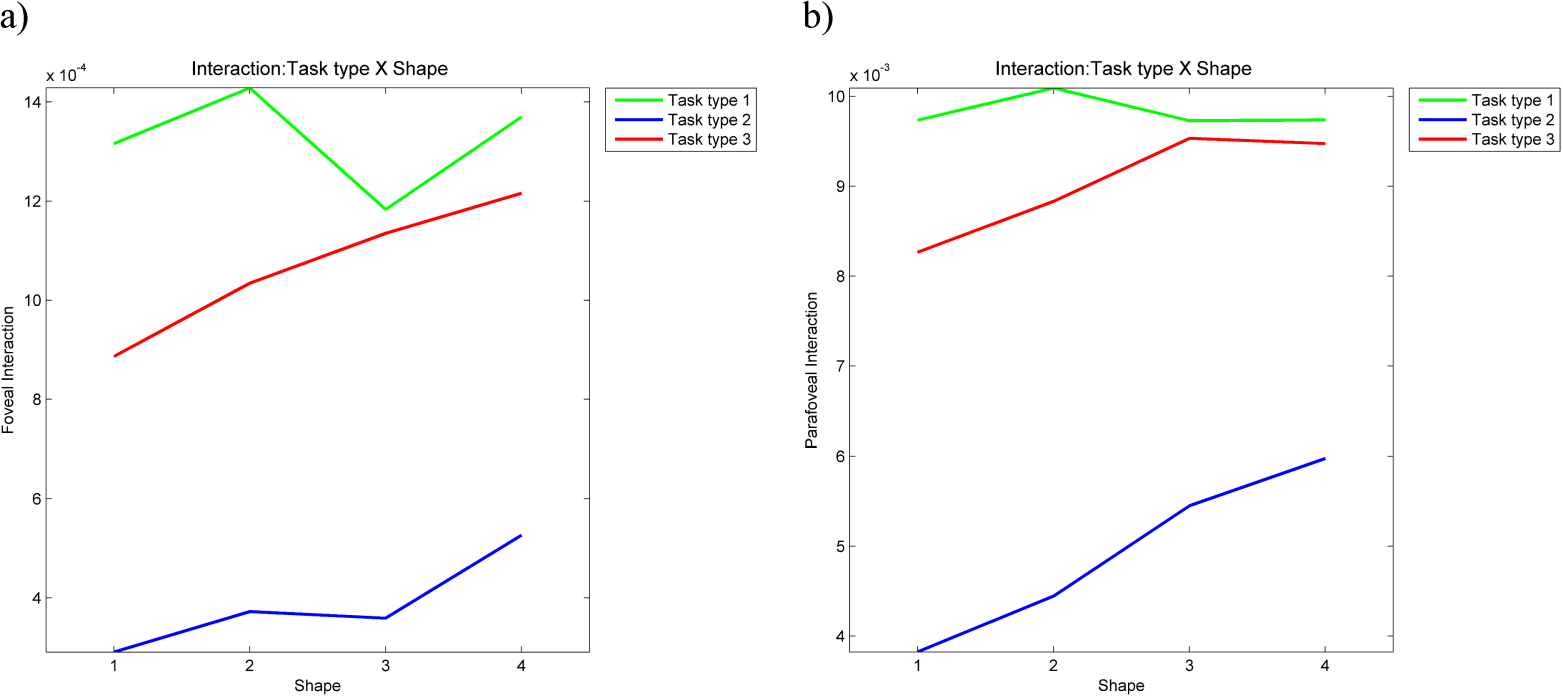
ANOVA plots for the foveal and parafoveal dynamic interaction measures.

The ANOVA of foveal dynamic interaction showed a highly significant main effect of task type (F = 36.3164, p = 4.8071*10^−7^<0.001), a significant main effect of shape type (F = 3.6593, p = 0.0247<0.05) and no significant effect of statistical interaction (F = 1.8896, p = 0.0994>0.05). The ANOVA on parafoveal dynamic interaction showed a highly significant main effect of task type (F = 53.6080, p = 2.6213*10^−8^<0.001), a significant main effect of shape type (F = 5.5663, p = 0.0042<0.05) and no significant effect of statistical interaction (F = 1.8122, p = 0.1140>0.05). Fig 16 shows ANOVA plots for foveal and parafoveal dynamic interaction respectively. In both plots, the lowest mean dynamic interaction is in task type 2 (eyes-first), which confirms our expectation that as this would be the most difficult task to perform (i.e., this is the task where we expected to see the lowest level of dynamic interaction between the gaze and the mouse). Surprisingly however, in both cases, the highest mean dynamic interaction occurs in task type 1 (natural tracing) rather than in task type 3 (mouse-first), which contradicts our expectation that there would be the smooth pursuit type of eye movement in task type 3 and consequently the highest level of dynamic interaction.

### Analysis Step 4. Linking time and interaction

Table 5 shows correlation coefficients between time spent on task and the levels of foveal and parafoveal dynamic interaction respectively. We calculated these two values per task and found that for task type 1 (natural tracing tasks, i.e., task numbers 1, 4, 7 and 10), task times seem to be relatively highly correlated with the dynamic interaction levels. This is however not so for the other two task types (eyes-first, mouse-first); perhaps suggesting that there is a *natural* coupling between the eye and the mouse movements during route tracing, but this coupling breaks when a participant tries to use the gaze or the mouse intentionally (consciously) in a particular way. Since both eyes-first and mouse-first task types require the participants to use the parafoveal (or even peripheral) vision to some degree, we might be documenting an instance of covert attention. This is an interesting finding, and upon further testing to confirm, it is imaginable that it could serve as an additional metric in eye tracking studies to understand whether the participants might be working with covert attention.

**Table 5:** Correlation coefficients between time on task and the two types of dynamic interaction. Note that natural tracing tasks have high correlations between task times and foveal/parafoveal interactions (shown on grey fields).

Task No.	Shape	(Task type #) Tracing method	Correlation between time on task and:	
			foveal interaction	parafoveal interaction
1	Circle	(1) Natural	0.65003	0.63267
2	Circle	(2) Eyes-first	0.34971	0.27819
3	Circle	(3) Mouse-first	0.055692	-0.1871
4	Rectangle	(1) Natural	0.81515	0.77223
5	Rectangle	(2) Eyes-first	-0.47987	-0.1705
6	Rectangle	(3) Mouse-first	-0.22204	-0.28724
7	Curve	(1) Natural	0.76818	0.72107
8	Curve	(2) Eyes-first	-0.36521	-0.44811
9	Curve	(3) Mouse-first	-0.37321	-0.28992
10	Star	(1) Natural	0.81328	0.7165
11	Star	(2) Eyes-first	-0.24797	0.071006
12	Star	(3) Mouse-first	0.053052	0.11458

## Conclusions and discussion

In this paper we introduce a new analytical methodology for quantifying dynamic interaction between gaze and mouse movements in a route tracing experiment. The methodology is based on recent developments in computational movement analysis and visualisation within GIScience and Remote Sensing. We further make use of experiment designs based on the principles of experimental psychology and HCI. This paper is therefore one of the first attempts at interdisciplinary knowledge exchange between these disciplines (computational movement analysis, GIScience, Remote Sensing psychology, vision science and HCI).

Our first aim was the introduction of the novel analytical methodology for comparison of dynamic interaction of gaze and mouse trajectories. We successfully demonstrated how bespoke spatio-temporal methods can solve the problem of comparing two movement types that are very different in terms of physics, and how the proposed method allows processing the data that these two movement processes generate.

We further tested our method against established methods for dynamic interaction in movement ecology. Ecological indices measure interaction between two physically similar movement types as well as depend on specific ecological concepts, such as home range [41] and are therefore not suitable for gaze and mouse data. For this reason we did not evaluate our method against them on real eye and mouse data, but created data for three simulated scenarios with different levels of interaction. This pattern was identified with all methods.

We also performed a sensitivity analysis to the two method parameters (voxel and kernel size). We found that our measure is sensitive to both, but that it increases/decreases consistently with the parameter values and further that the pattern of interaction in the three simulated cases is consistent. This type of behaviour is typical for algorithms working on a discretised model of space-time, such as 3D raster algorithms [64].

Our second aim was to conduct a (pilot) study about the visuo-perceptual processes to better understand the coupling between the eye and hand movements through our experiment [38]. Although it is important to note that we primarily designed the experiment to obtain tracking data with different levels of dynamic interaction between the gaze and the mouse, so that the data could be used to demonstrate and evaluate our new methodology. Combining our dual purposes (to validate the proposed methodology and to conduct a pilot study to better understand the coupling between eye and hand movements), we designed the experiment tasks with hypotheses based on low-level characteristics of human vision [1,2,4]. The task types were defined based on the type of control that the participants had over their eye movement, either conscious or subconscious (we assumed that the mouse movement would be always conscious in this case, because the task is actively tracing something) and were expected to produce data exhibiting a particular level of dynamic interaction in each task. In particular, we expected the highest level of interaction in the task type that was mimicking the smooth pursuit [31–33] (mouse-first, task type 3) and the lowest level of interaction in the task type that required constant changes between central and parafoveal/peripheral vision [20, 29] (eyes-first, task type 2), while we expected the level of interaction to be ‘in-between’ for the natural task (task type 1, the only one where eyes were not controlled consciously).

Interestingly, our results do not show what we expected, and do not corroborate previous propositions [19, 20]. We found the highest level of interaction when the tracing was ‘natural’ (task type 1), and the second highest when the mouse was moved first (‘mouse-first, i.e. task type 3), suggesting that perhaps smooth pursuit is difficult when the person who is using this mode of vision is also controlling the moving object indirectly, that is, when the object of attention (the mouse pointer) is displayed on the screen, rather than being held in the hand and looked at directly [31–33]. We speculate that perhaps it is not fully possible or at least very difficult to consciously perform ‘smooth pursuit’ for an object on the screen (the mouse pointer) that is moved indirectly through the use of another object (the mouse) manipulated by the hand. It is beyond the scope of this paper to further explore this effect, but we note that it could constitute an interesting follow-up research question.

When participants were asked to move their eyes first (task type 2) the gaze and mouse trajectories exhibit the lowest level of dynamic interaction, as expected based on previous work [20,29]. While we need further testing to confirm the presence of this effect under other conditions (e.g. for tasks different than route tracing and for various populations), this observation allows us to speculate that adding mouse movements in the mix of HCI measurement methods, and using our newly proposed dynamic interaction measure may be a promising way to determine when and how frequently a participant is operating using covert attention (consciously diverting their attention to an area other than their precise target).

Additionally, we found that both the task type (i.e. how to trace the object) and the geometric shape (what object are participants tracing) have an effect on the level of dynamic interaction, and both in terms of foveal and parafoveal attention.

We further employed a standard usability measure, the time spent on task, to investigate if this corresponds to the level of dynamic interaction for different task types [32–33]. Interestingly, we find high correlation in all natural tracing tasks, but low correlation in both eyes-first and mouse-first tasks. In other words, if the participants were not instructed to use their eyes first, or the mouse first to trace, the amount of time they use for completing the task correlate with the number of interactions between the mouse and gaze trajectories; while with the ‘conscious eye movements’ (eyes-first, or mouse-first) we see this coupling a lot less. This result may be suggesting that there is a natural coupling between the eye and the mouse movements when tracing the route without conscious thinking, but that this coupling breaks when a participant tries to use the gaze or the mouse intentionally (consciously) in a particular way. This is possibly explained by the fact that we told them to delay one of the actions (either tracing with their eyes or tracing with their mouse), which, for many participants, created an unnatural (forced) behaviour and affected some participants differently than others. For example, advancing age is a known factor for declining peripheral perception [65], thus in ‘natural’ tracing people use the foveal vision, and differences in the peripheral perception does not matter, while for the task types that requires the use of peripheral vision, they might have taken longer. Age also matters for motor skills, thus mouse use in an ‘unnatural task’ might have been slowing some participants down. Similarly, those who wear corrective glasses (often they are optimized for a specific part of the visual field, assuming forward vision) might have had to work ‘harder’ to complete the tasks that required the use of peripheral vision. However, these are just initial considerations based on our limited experiment and these results would need more dedicated research, where such factors are properly controlled.

By introducing a new analytical methodology for dynamic interaction of eye and mouse movements, we also aimed to test the claim that mouse trajectories could be used as a proxy for eye tracking data in evaluation studies. Based on our results, we suggest that for natural tracing tasks, which exhibit a high level of dynamic interaction, mouse-tracking could indeed give similar information as eye-tracking (and therefore be used as a proxy for attention). However, this can only be said for the specific conditions that we worked with: a tracing task (very different than e.g. free viewing or visual search), a clean stimulus without visual clutter, and relatively basic geometric shapes that are ‘closed’. The question remains if these findings can be replicated for situations where users are tracing routes on real maps, where a higher level of background visual clutter is present, or if the participants execute tasks that are fundamentally different than tracing. As mentioned in the description of our experiment, we intend to apply our new methodology to cases where route tracing is performed on more complex maps to investigate this as well as to other typical tasks in visual exploration of maps on the screen (e.g. free viewing, visual search).

In this study, we only considered the mouse as a pointing device on a static stimulus. The situation becomes much more complex when the display is dynamic and the mouse also acts as an interaction device with the on-screen visualisation, allowing various interactive operations to manipulate the visualisation (clicking, dragging, zooming, panning, etc.). Mouse tracking in such a dynamic visualisation environment was recently investigated using semantically enriched mouse trajectories [14], which are trajectories annotated with physical movement parameters (speed, direction, acceleration, type of mouse activity, etc.). We propose that in order to investigate eye and mouse coordination in such an environment, gaze trajectories could similarly be semantically enriched with standard eye tracking measures (e.g. number and duration of fixations, segmentation of a trajectory into fixations and saccades, etc.). Semantically enriched trajectories of both types could then be compared with relevant existing or new trajectory data mining methods. For example, in movement ecology, Papastamatiou et al. [66] create the so-called ‘activity seascapes’, which are space-time volumetric representations of animal trajectories annotated with the diurnal activity levels for each species, where the activity level is directly incorporated in the way the space-time volume is calculated.

Finally we would like to outline two main limitations of our study. Computationally, our methods are based on the volumetric division of space and time, creating new data sets that can be of considerable size. The size of these volumes is dependent on the size of the voxels in this division and the number of voxels in a certain volume increases with the third power of each new subdivision in each dimension. This means that the time for any algorithmical processing of these volumes also increases with the third power, which can lead to very long processing times for relatively small changes in voxel size. This limitation may be overcome either by rewriting the code using parallel processing or by using methods from computational geometry and 3D computer graphics, which strive to optimise computational complexity of volumetric algorithms [67].

The second limitation is related to the experiment and more specifically, its sample size: as this experiment was primarily designed to produce test data for our new methods, we recruited a limited number of participants. The results of our analysis on the connection between the eye and the mouse while tracing a route on the screen are therefore still somewhat speculative, as we outline above. This could be improved by performing larger experiments and indeed we propose that our experiment should serve as a starting point for more detailed future studies.

To conclude, this is, as far as we know, one of the first attempts to develop bespoke spatio-temporal movement analysis methods specifically for an HCI context and we hope that the paper will raise awareness in the HCI community of the possibilities that such cross-disciplinary exchange can bring. On the other hand, our new dynamic interaction quantification methodology is general (apart from linking the density parameters to the characteristics of human vision and foveal/parafoveal attention) and could thus be used in any other situation that requires comparison of two different types of simultaneous movement. There is therefore also scope of further cross-disciplinary knowledge transfer into other disciplines that observe and measure movement, such as movement ecology or analysis of human mobility.

## Supporting information

S1 Code R implementation of the new dynamic interaction measure. (ZIP)

S1 Data Anonymised gaze and mouse trajectories for ten participants in twelve tracing tasks. (ZIP)

S2 Data Simulated trajectories used in method comparison and sensitivity analysis. (ZIP)
